# Direct current stimulation enhances neuronal alpha-synuclein degradation in vitro

**DOI:** 10.1038/s41598-021-81693-8

**Published:** 2021-01-26

**Authors:** Gessica Sala, Tommaso Bocci, Valentina Borzì, Marta Parazzini, Alberto Priori, Carlo Ferrarese

**Affiliations:** 1 Laboratory of Neurobiology, NeuroMI - Milan Center for Neuroscience, School of Medicine and Surgery, University of Milano-Bicocca, via Cadore, 48, 20900 Monza, MB Italy; 2 Centro “Aldo Ravelli” per le Neurotecnologie e le Terapie Neurologiche Sperimentali, Dipartimento di Scienze della Salute, Università degli Studi di Milano and ASST Santi Paolo e Carlo, Milan, Italy; 3 Istituto di Elettronica e di Ingegneria Dell’Informazione e delle Telecomunicazioni (IEIIT), Consiglio Nazionale delle Ricerche (CNR), Milan, Italy; 4 Department of Neurology, ASST-Monza, San Gerardo Hospital, Monza, Italy

**Keywords:** Diseases, Neurology, Pathogenesis

## Abstract

Despite transcranial Direct Current Stimulation (DCS) is currently proposed as a symptomatic treatment in Parkinson’s disease, the intracellular and molecular mechanisms elicited by this technique are still unknown, and its disease-modifying potential unexplored. Aim of this study was to elucidate the on-line and off-line effects of DCS on the expression, aggregation and degradation of alpha-synuclein (asyn) in a human neuroblastoma cell line under basal conditions and in presence of pharmachologically-induced increased asyn levels. Following DCS, gene and protein expression of asyn and its main autophagic catabolic pathways were assessed by real-time PCR and Western blot, extracellular asyn levels by Dot blot. We found that, under standard conditions, DCS increased monomeric and reduced oligomeric asyn forms, with a concomitant down-regulation of both macroautophagy and chaperone-mediated autophagy. Differently, in presence of rotenone-induced increased asyn, DCS efficiently counteracted asyn accumulation, not acting on its gene transcription, but potentiating its degradation. DCS also reduced intracellular and extracellular asyn levels, increased following lysosomal inhibition, independently from autophagic degradation, suggesting that other mechanisms are also involved. Collectively, these findings suggest that DCS exerts on-line and off-line effects on the expression, aggregation and autophagic degradation of asyn, indicating a till unknown neuroprotective role of tDCS.

## Introduction

In the last 20 years transcranial Direct Current Stimulation (tDCS) has emerged as a non-invasive, and safe technique to modulate neuronal excitability both in healthy subjects and patients in a range of diseases including stroke, psychiatric conditions and movement disorders. In Parkinson’s disease (PD) tDCS has been shown to improve motor and cognitive performance^1–7^. More important, tDCS has been demonstrated to improve both speech and axial disturbances, whereas even invasive brain stimulation failed to induce significant changes^8,9^.

Although the literature data on the clinical benefits of tDCS are abundant, the exact mechanisms of action of this technique has not yet been fully elucidated, both at the cellular and molecular level; in particular, it remains to be determined whether tDCS is able to interfere with gene expression and protein folding, whether the tDCS-induced neuronal activity modulation occurs during (on-line effects) or following stimulation (off-line effects), and how long the effects of tDCS persist. Moreover, to date tDCS has been proposed as a symptomatic treatment, without exploring its disease-modifying potential. A more profound impact on PD pathogenesis is suggested by recent papers showing that tDCS enhances the integration and survival of dopaminergic cell transplants in a rat model^10^.

Aim of this study was to elucidate the molecular effects of the direct current stimulation (DCS) on the expression, aggregation and degradation of alpha-synuclein (asyn). To this aim, we used a human neuroblastoma cell line SH-SY5Y, expressing a dopaminergic phenotype and widely used to create PD models^11^, to reproduce in vitro the parameters (current intensity and time) of stimulation used in the clinical practice in PD patients underwent tDCS. We firstly assessed the on-line—immediately at the end of stimulation—and off-line—1 and 17 h following stimulation—effects of DCS on the expression of monomeric and oligomeric/aggregated forms of asyn in cells under standard culture conditions. Considering the role of autophagy in the catabolism of asyn and the knowledge that this degradative system is impaired and contributes to PD pathogenesis^12,13^, the effects of DCS was investigated on the activity of the 2 main autophagic pathways, macroautophagy and chaperone-mediated autophagy (CMA). In the second part of the study, the effects of DCS on asyn protein and gene expression and degradation were evaluated in 2 different in vitro models of synucleinopathy obtained by treating the same cell line with a mitochondrial or a lysosomal inhibitor causing an accumulation of asyn.

## Results

### Effect of direct current stimulation (DCS) in SH-SY5Y cells under basal conditions

The effects of 1 mA DCS on asyn expression and its degradative pathways were assessed in human neuroblastoma SH-SY5Y cells at 3 different time points from stimulation: Recovery 0 (R0) corresponding to cells collected at the end of 20 min stimulation, R1 and R17, corresponding to cells collected 1 and 17 h after the end of the stimulation, respectively. This experimental design was chosen in order to analyze the on-line (R0, immediately at the end of the stimulation) and off-line (R1 and R17) effects of DCS on SH-SY5Y cells.

#### DCS does not affect cell morphology and viability

We initially verified that the protocol of DCS used in this study was well-tolerated by cell cultures, which were overtime maintained in a stable environment with a physiological temperature (37 °C) and a controlled atmosphere (5%CO_2_, about 95% relative humidity).

The images of cells captured at the different time points after DCS indicated that this treatment does not alter the physiological cell morphology, although the mechanic effect of sponges placed in the Petri dishes caused a partial detachment of cells under the sponges; this effect was especially appreciable in sham (cells underwent the experimental setup with no current passage) and R0, with a progressive normalization of the cell layer homogeneity overtime, as a consequence of the physiological cell growth. No change in cell viability was found at any time points in cells exposed to DCS with respect to sham (Supplementary Fig. 1).

#### DCS modulates the expression and the aggregation status of asyn

The effects of DCS was assessed on gene and protein expression of asyn, specifically analyzing the different asyn protein isoforms: soluble/monomeric form (19 kDa) and oligomeric/aggregated forms (about 50 and 100 kDa).

**Figure 1:**
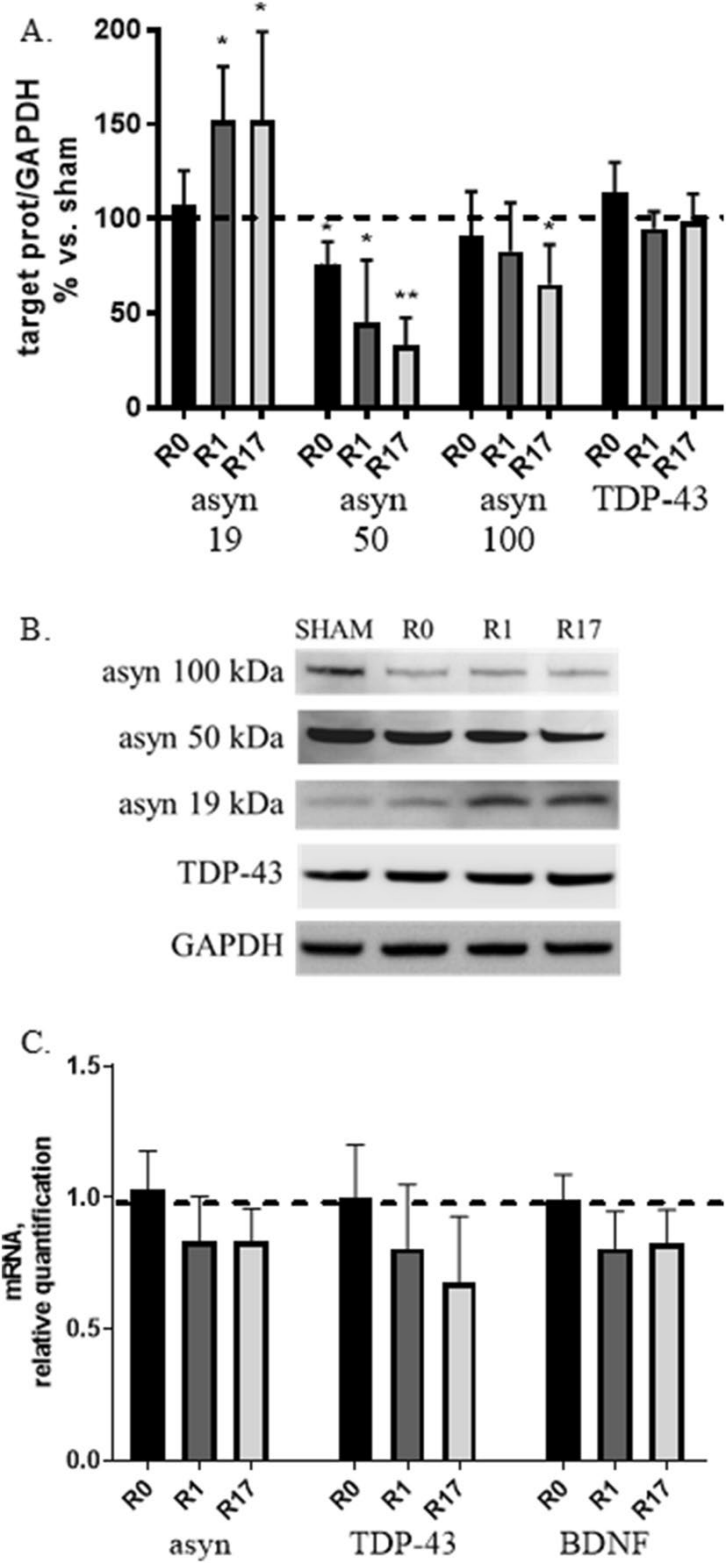
Effects of DCS on protein (**A**,**B**) and gene (**C**) expression of asyn, TDP-43 and BDNF (mRNA levels only) in SH-SY5Y cells under basal conditions at the end of stimulation (R0), after 1 and 17 h (R1 and R17). Reduced oligomeric/aggregated and increased monomeric asyn forms were found in DCS-treated cells (**A**,**B**). (**B**) Representative Western blot image showing immunoreactivity at different time points (R0, R1 and R17) for monomeric (19 kDa) and oligomeric/aggregated (50 and 100 kDa) asyn forms, TDP-43 and GAPDH, used as internal standard, in cells exposed to DCS or sham. Full-length blots are presented in Supplementary M&M. (N = 3; **p < 0.01, *p < 0.05 vs. sham).

Western blot analyses indicated that DCS induces a significant increase of monomeric asyn at R1 and R17 (+ 50%, p < 0.05), a reduction of oligomeric (50 kDa) asyn at all analyzed time points (− 25% and − 55%, p < 0.05 at R0 and R1 respectively; − 65%, p < 0.01 at R17) and a progressive reduction of 100 kDa asyn that becomes significant at R17 (− 35%, p < 0.05) (Fig. 1A,B). The quantification of asyn gene expression by real-time qPCR evidenced no significant change in asyn mRNA levels after DCS, although a mild reduction was observed both at R1 and R17 (Fig. 1C). These results indicate that DCS, applied to cells under standard culture conditions, alters the expression of the different asyn isoforms, shifting their balance towards the monomeric form and reducing the oligomeric forms.

To verify the specificity of the effects of DCS on asyn, the expression of TDP-43 (TAR DNA binding protein-43), the protein that accumulates in motor neurons of patients affected by amyotrophic lateral sclerosis (ALS) or frontotemporal dementia (FTD), was also quantified. No effect of DCS was evidenced on TDP-43 protein expression (Fig. 1A,B), while TDP-43 gene expression showed a trend similar to that observed for asyn (Fig. 1C).

Finally, the effects of DCS were also assessed on the gene transcription of BDNF (Brain-Derived Neurotrophic Factor), a neurotrophin essential for neuronal development and survival, synaptic plasticity and energy homeostasis, whose levels are reduced in both nigral neurons^14^ and peripheral blood^15^ from PD patients. In this study, BDNF, based on the ability to up-regulate its transcription under stress conditions, was used as a ‘molecular sensor’ of the overall cellular homeostasis. The quantification of BDNF mRNA levels showed that DCS does not induce BDNF transcription, whose levels are unaltered at R0 and even slightly reduced at R1 and R17 (Fig. 1C), consistent with the view that DCS, applied to cells under standard culture conditions, does not perturb, but even tend to improve, the cellular homeostasis.

#### DCS down-regulates autophagic pathways under basal conditions

The effects of DCS on the functionality of the two main autophagic pathways—macroautophagy and chaperone-mediated autophagy (CMA)—responsible for asyn catabolism were also evaluated.

Beclin-1, a protein involved in the early stages of phagophore formation, LC3 (Light Chain 3), which in its lipidated form (LC3-II) represents a marker of autophagosomes, and p62, substrate of the macroautophagy, were chosen as markers of macroautophagy.

**Figure 2:**
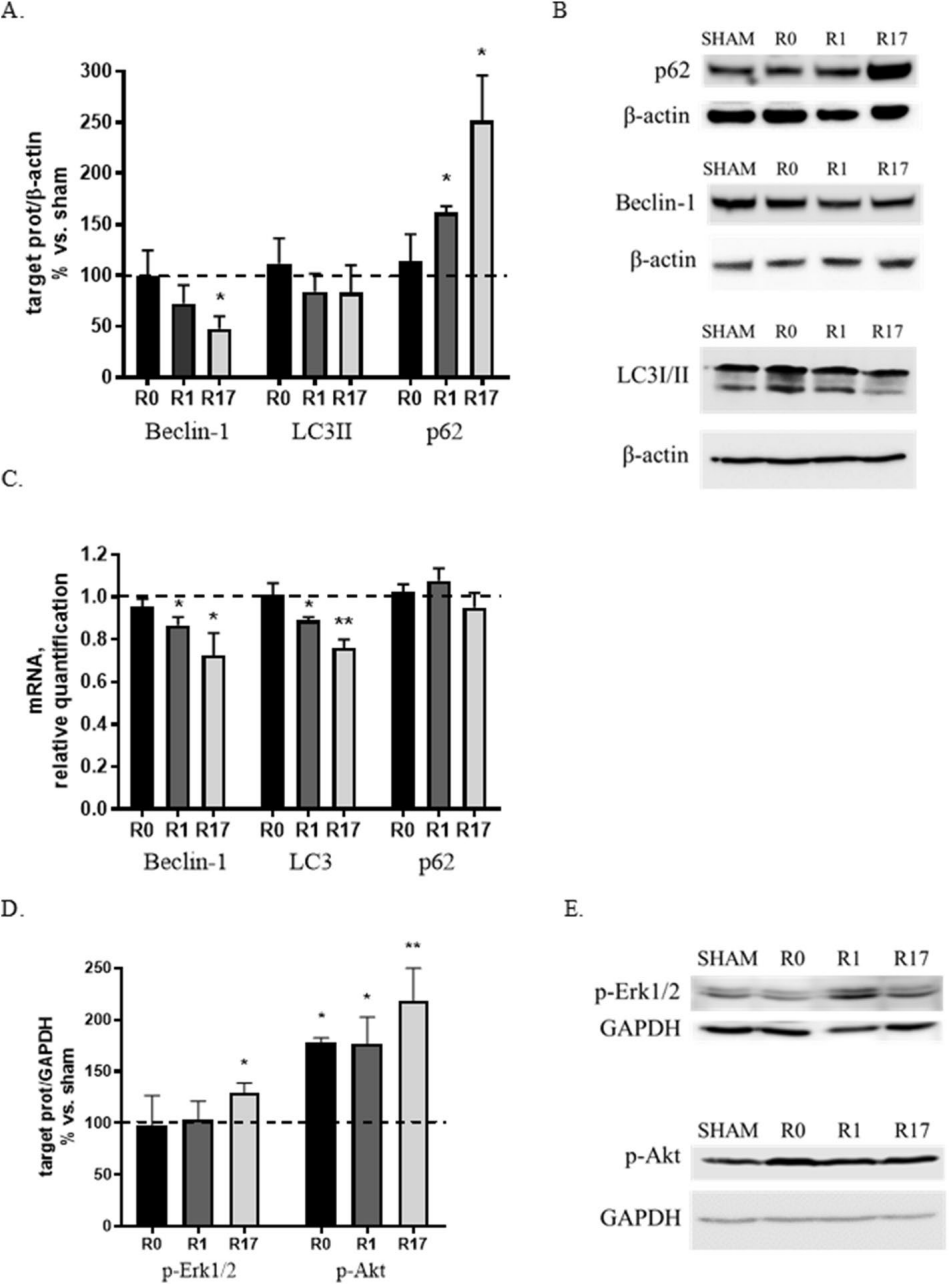
Effects of DCS on protein and gene expression of macroautophagy markers (Beclin-1, LC3, p62) (**A**,**C**) and on the activation/phosphorylation of 2 regulatory pathways (Erk1/2, Akt) (**D**,**E**) in SH-SY5Y cells under basal conditions at the end of stimulation (R0), after 1 and 17 h (R1 and R17). A down-regulation of macroautophagy was found in DCS-treated cells. (**B**) Representative Western blot image showing immunoreactivity at different time points (R0, R1 and R17) for p62, Beclin-1, LC3 and β-actin, used as internal standard, in cells exposed to DCS or sham. Full-length blots are presented in Supplementary M&M. (**E**) Representative Western blot image showing immunoreactivity at different time points (R0, R1 and R17) for phosho-Akt, phospho-Erk1/2 and GAPDH, used as internal standard, in cells exposed to DCS or sham. Full-length blot for phospho-Akt and available cropped blot for phospho-Erk1/2 are presented in Supplementary M&M. (N = 3; **p < 0.01, *p < 0.05 vs. sham).

Protein levels of Beclin-1 and LC3-II appeared unchanged at the end of the stimulation (R0) and showed a tendency to decrease at R1 and R17, which reached statistical significance for Beclin-1 (− 50%, p < 0.05) at R17 (Fig. 2A,B). Following DCS, a progressive increase in the levels of p62 was observed, with a significant increase at R1 (+ 60%, p < 0.05) and R17 (+ 150%, p < 0.01) (Fig. 2A,B). A reduction of Beclin-1 mRNA levels was found at R1 and R17 (-15 and -30%, p < 0.05, respectively). Similar results were obtained for LC3, with a significant decrease in gene transcription at R1 (− 12%, p < 0.05) and R17 (− 25%, p < 0.01) (Fig. 2C). No change was evidenced in p62 mRNA levels after DCS (Fig. 2C).

Collectively, the observed increase of the substrate p62 and the decrease of the effectors Beclin-1 and LC3 indicate that DCS applied to cells under standard culture conditions results in a time-dependent down-regulation of macroautophagy.

The activation levels—corresponding to the state of phosphorylation—of Erk 1/2 and Akt, 2 regulatory kinases of macroautophagy acting as inducers of mTOR, the main negative regulator of macroautophagy, were also analyzed.

Following DCS, a 30% increase (p < 0.05) in phospho-Erk 1/2 levels was observed at R17, as well as a marked increase in phospho-Akt levels at all analyzed times (+ 80%, p < 0.05 at R0 and R1 and + 120%, p < 0.01 at R17) (Fig. 2D,E). No effect of DCS was evidenced on total Erk 1/2 and Akt expression. The marked increase in Akt phosphorylation suggests that this pathway, rather than Erk 1/2 pathway, may play a major inhibitory role on macroautophagy through the modulation of mTOR.

**Figure 3:**
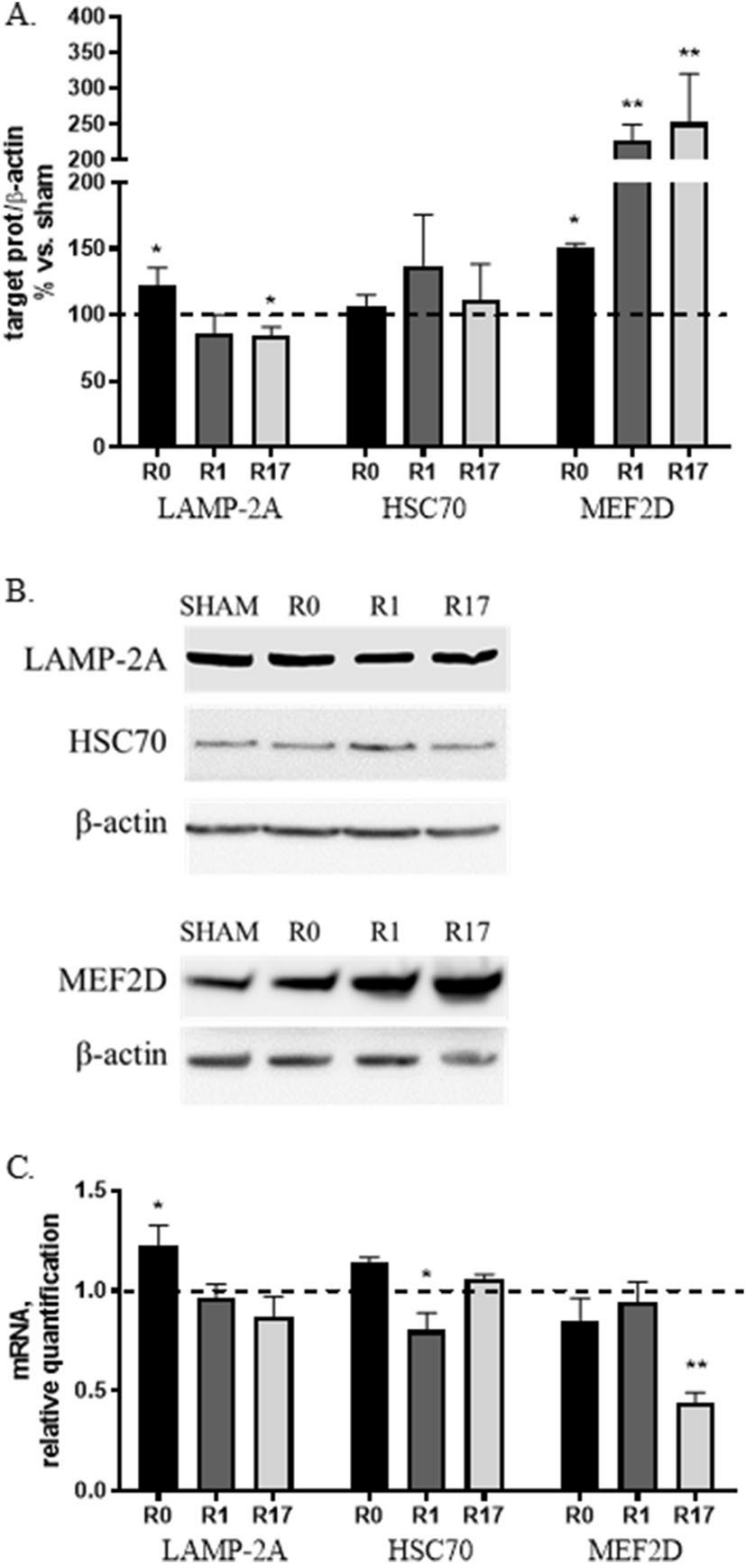
Effects of DCS on protein (**A**,**B**) and gene (**C**) expression of CMA markers (LAMP-2A, HSC70, MEF2D) in SH-SY5Y cells under basal conditions at the end of stimulation (R0), after 1 and 17 h (R1 and R17). A down-regulation of CMA was found in DCS-treated cells. (**B**) Representative Western blot image showing immunoreactivity at different time points (R0, R1 and R17) for LAMP-2A, HSC70, MEF2D and β-actin, used as internal standard, in cells exposed to DCS or sham. Full-length blot for MEF2D and available cropped blot for LAMP-2A and HSC70 are presented in Supplementary M&M. (N = 3; **p < 0.01, *p < 0.05 vs. sham).

LAMP-2A (Lysosome-associated Membrane Protein 2A), the lysosomal receptor, HSC70 (Heat Shock Cognate Protein 70), the carrier protein, and the substrate MEF2D (Myocyte enhancer factor 2D) were evaluated as markers of CMA. A modest increase (+ 25%, p < 0.05) of LAMP-2A protein levels was observed at R0, while a mild reduction emerged at R17 (− 23%, p < 0.05). No significant change was found in HSC70 protein levels, although a tendency to increased levels was observed at R1 (Fig. 3A,B). A marked increased of MEF2D protein levels was found at all investigated time points (+ 50%, p < 0.05 at R0; + 120% and + 150%, p < 0.01 at R1 and R17 respectively) (Fig. 3A,B). An increase of LAMP-2A mRNA levels (+ 22%, p < 0.05) at R0 and a reduction of HSC70 mRNA levels (− 20%, p < 0.05) at R1 were found. Furthermore, a marked reduction of MEF2D gene expression (− 55%, p < 0.01) was observed at R17 (Fig. 3C).

Taken together, these results—increase of the substrate MEF2D and tendency to a decrease of the lysosomal receptor LAMP-2A—are consistent with a time-dependent inhibitory effect on CMA of DCS applied to cells under standard culture conditions, similarly with what evidenced for macroautophagy.

#### DCS does not influence extracellular levels of soluble asyn under basal conditions

Dot blot analyses performed on culture cell media collected 17 h after DCS (R17) evidenced no change in soluble extracellular asyn levels in stimulated vs. sham media, indicating that DCS under basal condition does not influence the release of soluble asyn (data not shown).

### Effect of direct current stimulation (DCS) in 2 in vitro models of synucleinopathy

**Figure 4:**
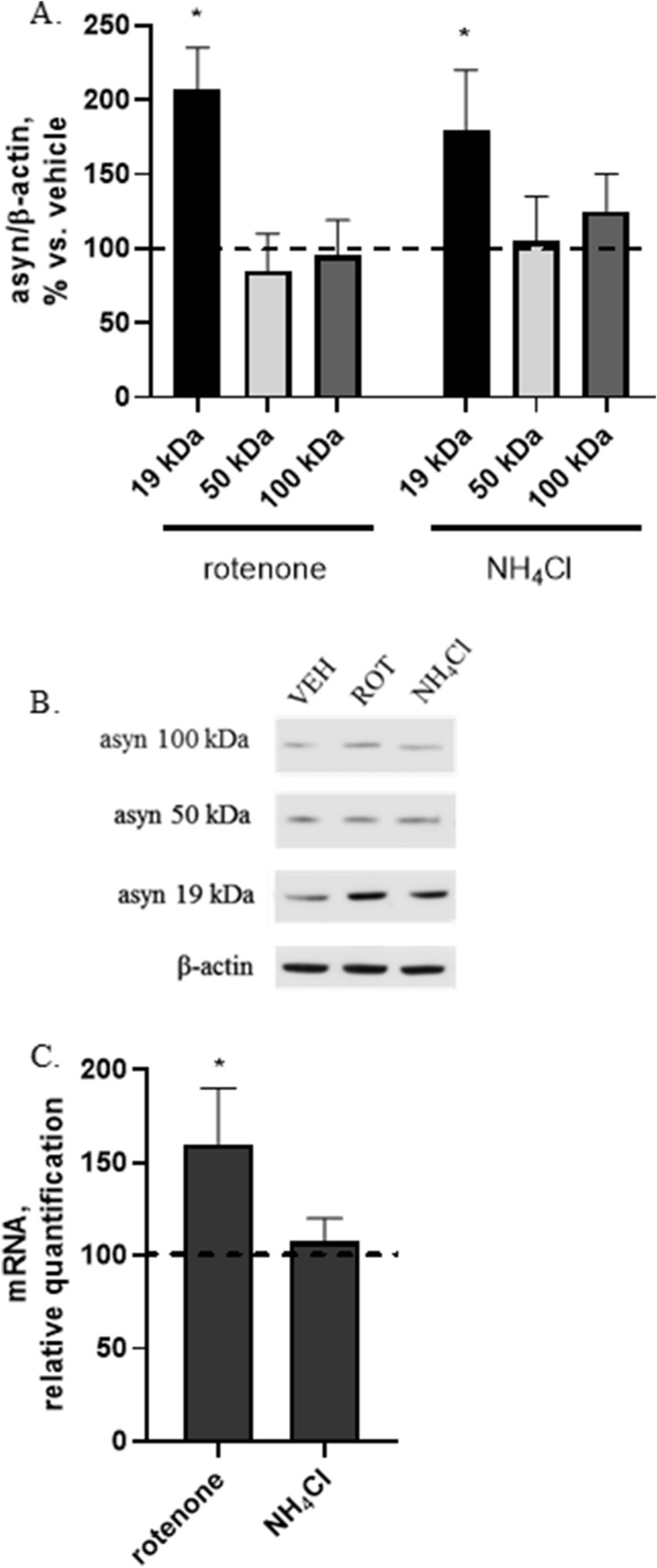
Effect of rotenone (400 nM, 24 h) or NH_4_Cl (10 mM, 24 h) on protein expression of monomeric (19 kDa) and oligomeric/aggregated (50 and 100 kDa) asyn forms (**A**) and asyn gene expression (**C**). Increased monomeric asyn was found in rotenone- or NH_4_Cl-treated cells (**A**,**B**) and increased asyn gene transcription was found in rotenone-treated cells (**C**). (**B**) Representative Western blot image showing immunoreactivity for monomeric (19 kDa) and oligomeric/aggregated (50 and 100 kDa) asyn forms and β-actin, used as internal standard, in vehicle-treated cells (VEH) and cells exposed to rotenone (400 nM, 24 h) or NH_4_Cl (10 mM, 24 h). Full-length blot is presented in Supplementary M&M. (N = 3; *p < 0.05 vs. vehicle).

To induce an intracellular increase of asyn, SH-SY5Y cells were treated with rotenone, a mitochondrial complex I inhibitor used to create in vivo and in vitro PD models with neuronal degeneration and asyn aggregate formation, or ammonium chloride (NH_4_Cl), an inhibitor of lysosomal proteases causing asyn accumulation. Concentration and time exposure for both compounds were chosen based on previous published results from our group on the same cell line^16,17^, and the effect of these treatments on asyn protein and gene expression verified. Exposure to 400 nM rotenone for 24 h induced a significant increase of monomeric asyn (+ 100%, p < 0.05), with no change in oligomeric/aggregated forms (Fig. 4A,B), associated with an increased asyn gene expression (+ 60%, p < 0.05) (Fig. 4C). A similar increase of monomeric asyn (+ 80%, p < 0.05) was also observed after 24 h treatment with 10 mM NH_4_Cl (Fig. 4A,B), but, differently from what reported following rotenone, no change in asyn mRNA levels was found (Fig. 4C).

Both compounds were therefore able to favor an in vitro an accumulation of intracellular asyn typical of synucleinopathies.

#### DCS counteracts the rotenone-induced asyn increase

To assess the effect of DCS on asyn expression in rotenone-treated cells the following experimental protocol was used: after exposure to 400 nM rotenone for 24 h, medium containing rotenone was replaced with fresh standard medium, cells underwent DCS (1 mA, 20 min) and were collected after 17 h (R17).

**Figure 5:**
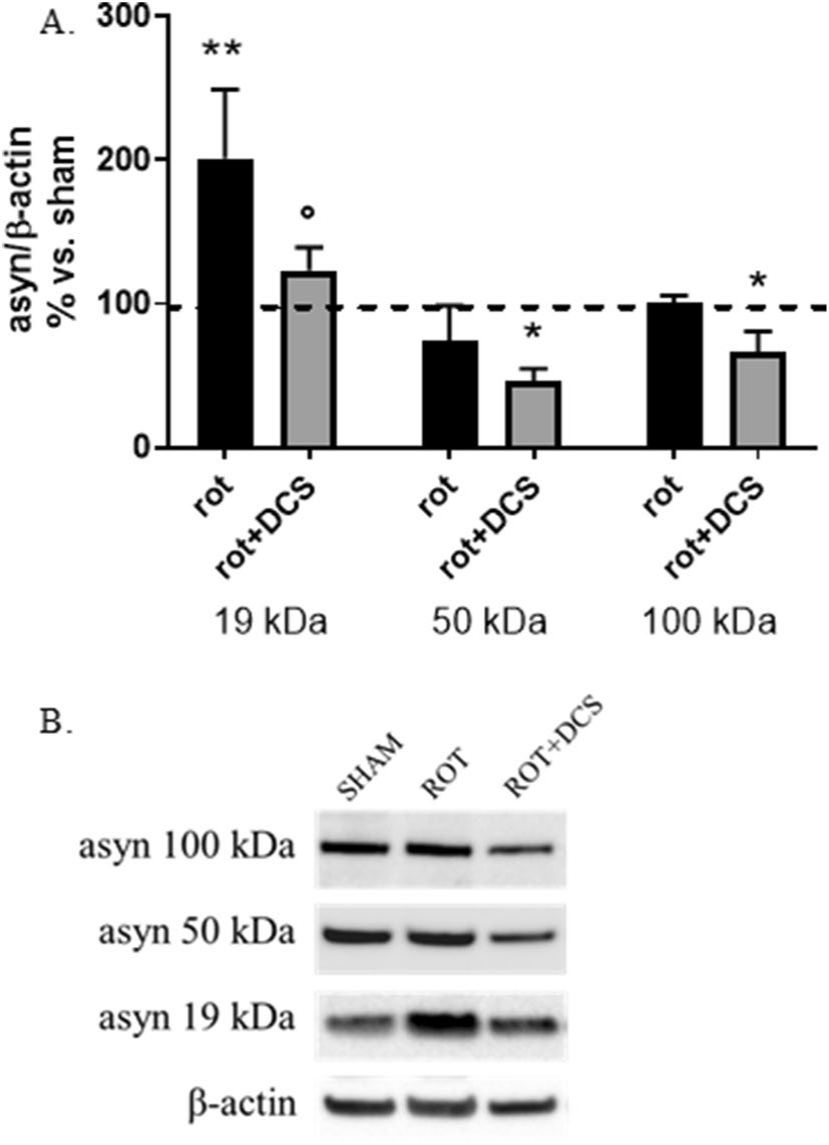
Effects of DCS on protein expression of monomeric (19 kDa) and oligomeric/aggregated (50 and 100 kDa) asyn forms in cells treated with rotenone (400 nM, 24 h); cells were collected 17 h after DCS (R17); the effect of rotenone alone on asyn was also shown. DCS counteracted the rotenone-induced increase of monomeric asyn and reduced oligomeric/aggregated asyn forms (**A**,**B**). (**B**) Representative Western blot image showing immunoreactivity for monomeric (19 kDa) and oligomeric/aggregated (50 and 100 kDa) asyn forms and β-actin, used as internal standard, in cells exposed to sham and cells treated with rotenone alone or rotenone and DCS. Full-length blot is presented in Supplementary M&M. (N = 3; **p < 0.01, *p < 0.05 vs. untreated cells, °p < 0.05 vs. rotenone-treated cells).

As expected and in line with results described in Fig. 4A, rotenone caused an increase (+ 100%, p < 0.01) of monomeric asyn levels with no change in oligomeric forms (Fig. 5A,B). Interestingly, DCS was able to significantly counteract the rotenone-induced increase in monomeric asyn, by decreasing its levels almost to control values (− 80% vs. rotenone-treated cells, p < 0.05). Although rotenone alone did not modify the levels of oligomeric asyn forms with respect to untreated cells, after DCS a significant reduction (− 55 for asyn 50 kDa and − 35% for asyn 100 kDa vs. untreated cells, p < 0.05) of oligomeric asyn forms was also observed (Fig. 5A,B). No effect of DCS was found on asyn gene expression in rotenone-treated cells, that show mRNA levels similar to cells exposed to rotenone alone (data not shown, effect of rotenone alone shown in Fig. 4B).

These results indicate that DCS counteracts the intracellular increase of asyn induced by rotenone, not through a decrease of its gene transcription, but likely potentiating its degradation.

#### DCS up-regulates autophagic pathways in rotenone-treated cells

To verify the hypothesis that, in the rotenone-induced synucleinopathy in vitro model, DCS can potentiate the activity of the 2 main autophagic pathways involved in asyn degradation, the protein levels of macroautophagy and CMA markers were analyzed.

**Figure 6:**
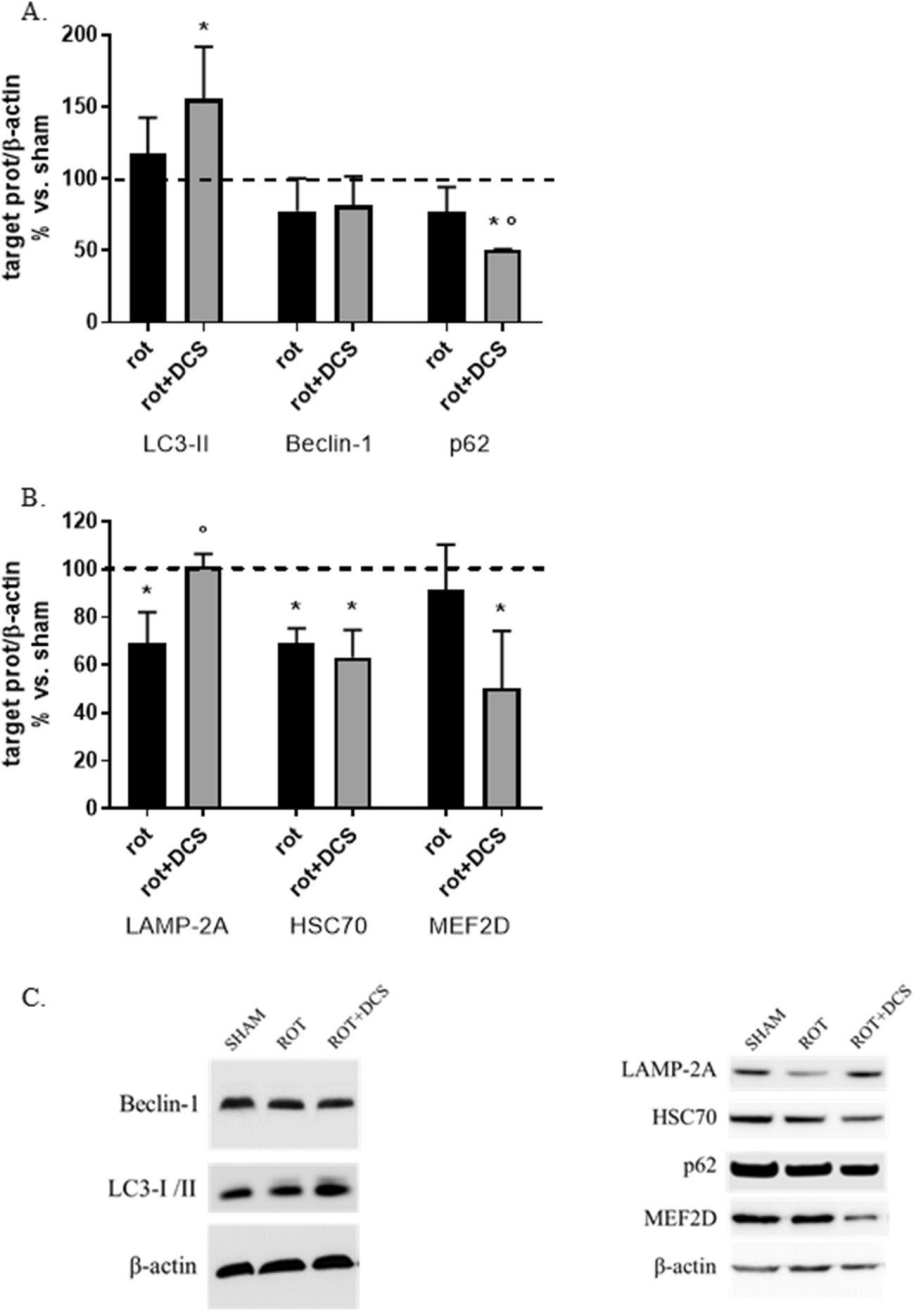
Effects of DCS on protein expression of macroautophagy (**A–C**) and CMA (**B**,**C**) markers in cells treated with rotenone (400 nM, 24 h); cells were collected 17 h after DCS (R17). An up-regulation of macroautophagy (**A–C**) and CMA (**B**,**C**) was found in rotenone-treated cells after DCS. (**C**) Representative Western blot images showing immunoreactivity for Beclin-1, LC3-I/II, LAMP-2A, HSC70, p62 and MEF2D and β-actin, used as internal standard, in sham-treated cells and cells exposed to rotenone alone or rotenone and DCS. Full-length blot or details on the used methodological approach are included in Supplementary M&M. (N = 3; *p < 0.05 vs. untreated cells, °p < 0.05 vs. rotenone-treated cells).

A significant increase (+ 50%, p < 0.05 vs. untreated cells) in the autophagosome marker LC3-II and a reduction (− 50%, p < 0.05 vs. untreated and rotenone-treated cells) of the substrate p62 were observed in rotenone-treated cells underwent DCS (Fig. 6A–C). No effect of DCS was evidenced in Beclin-1 protein levels (Fig. 6A–C).

Considering that rotenone caused a 30% reduction (p < 0.05) of both the CMA lysosomal receptor LAMP-2A and the carrier protein HSC70, DCS was able to significantly increase LAMP-2A protein levels with respect to rotenone-treated cells (+ 30%, p < 0.05), with no effect on HSC70 expression. A 50% decrease of the substrate MEF-2D was also evidenced in cells underwent DCS (Fig. 6B,C).

These results support the view that, in rotenone-treated cells, DCS leads to an up-regulation of both macroautophagy and CMA.

#### DCS counteracts the NH_4_Cl-induced asyn increase also independently of autophagic degradation

In the last paragraphs we described that DCS is able to counteract rotenone-induced increase of asyn through an up-regulation of asyn autophagic degradation. Now, to verify if the potentiation of autophagy is the only mechanism possibly involved in this effect of DCS, we treated cells with the lysosomal inhibitor ammonium chloride to block both macroautophagy- and CMA-dependent asyn degradation. Cells were treated with 10 mM NH_4_Cl for 24 h and, during this time (7 h after the begin of the treatment) and always in presence of the inhibitor, cells underwent DCS (1 mA, 20 min) and were finally collected after 17 h (R17).

**Figure 7:**
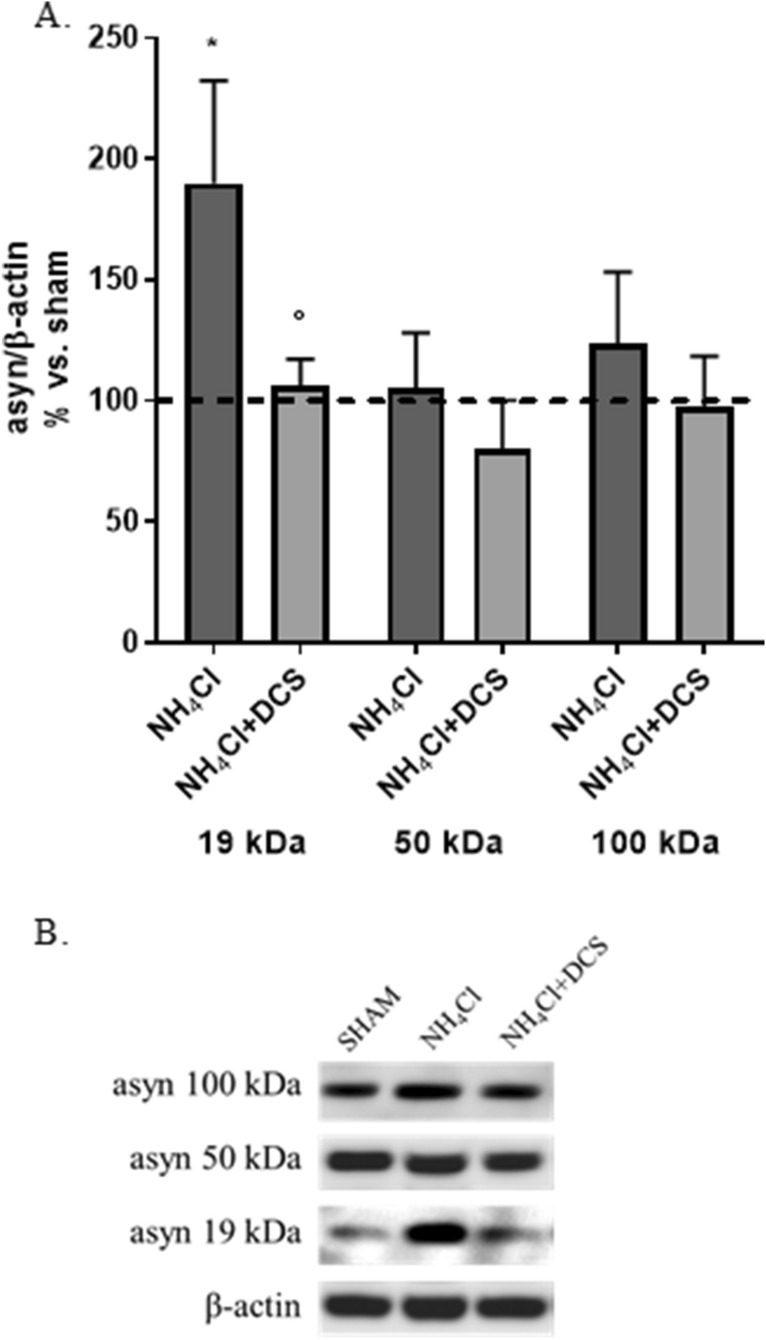
Effects of DCS on protein expression of monomeric (19 kDa) and oligomeric/aggregated (50 and 100 kDa) asyn forms in cells treated with NH_4_Cl (10 mM, 24 h); cells were collected 17 h after DCS (R17); the effect of NH_4_Cl alone on asyn was also shown. DCS counteracted the NH_4_Cl-induced increase of monomeric asyn (**A**,**B**). (**B**) Representative Western blot image showing immunoreactivity for monomeric (19 kDa) and oligomeric/aggregated (50 and 100 kDa) asyn forms and β-actin, used as internal standard, in cells exposed to sham and cells treated with NH_4_Cl alone or NH_4_Cl and DCS. Blot details and available images are included in Supplementary M&M. (N = 3; *p < 0.05 vs. untreated cells, °p < 0.05 vs. NH_4_Cl-treated cells).

As expected and in line with results described in Fig. 4A, NH_4_Cl caused an increase (+ 80%, p < 0.05) of monomeric asyn levels with no change in oligomeric forms (Fig. 7A,B). Interestingly, DCS was able to significantly counteract the NH_4_Cl-induced increase in monomeric asyn (-75%, p < 0.05 vs. NH_4_Cl-treated cells) (Fig. 7A,B). Similarly to what observed in rotenone-treated cells, a trend to reduction of oligomeric asyn forms was also observed after DCS in NH_4_Cl-treated cells, although NH_4_Cl alone did not significantly affect the expression of these forms (Fig. 7A,B).

These results, obtained in condition of lysosomal inhibition, indicate that DCS reduces the increase of asyn protein levels caused by a block of its autophagic degradation also through other and autophagy-independent mechanisms.

#### DCS counteracts the NH_4_Cl-induced increase of extracellular levels of soluble asyn

**Figure 8:**
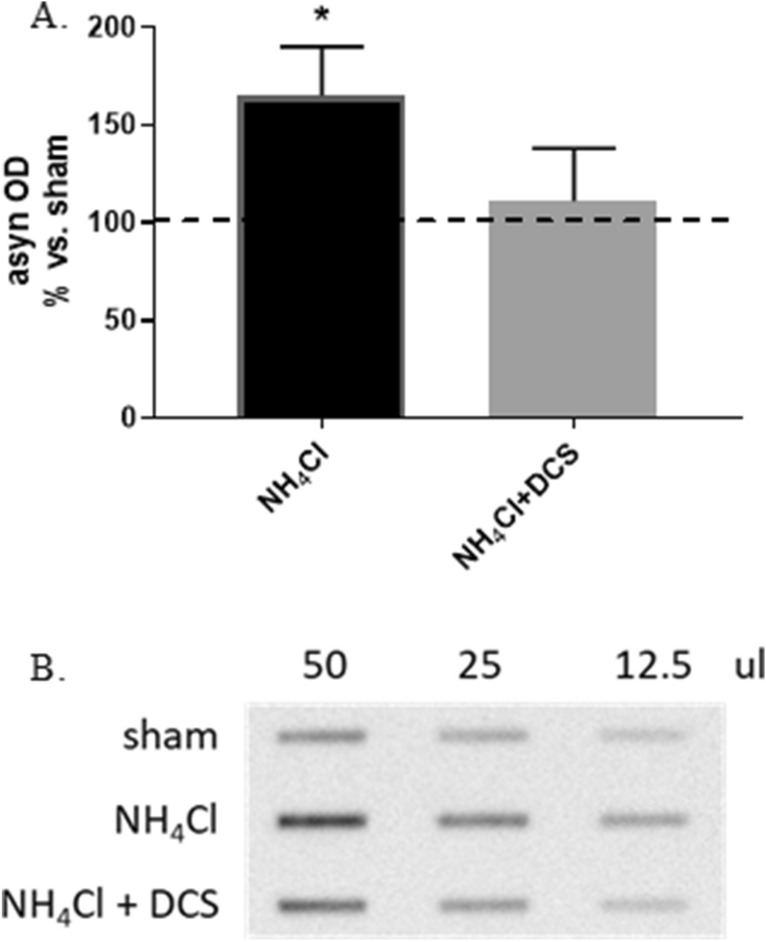
Effect of DCS on extracellular soluble asyn levels in culture media of cells treated with NH_4_Cl (10 mM, 24 h); culture media were collected 17 h after DCS (R17); the effect of NH_4_Cl alone on extracellular soluble asyn was also shown. DCS counteracted the NH_4_Cl-induced increase of extracellular levels of soluble asyn (**A**,**B**). (**A**) Histogram showing the levels of extracellular soluble asyn expressed as optical density (OD) in cells treated with NH_4_Cl alone or NH_4_Cl and DCS. (**B**) Representative Dot blot image showing immunoreactivity for extracellular soluble asyn in cells exposed to sham and cells treated with NH_4_Cl alone or NH_4_Cl and DCS (volume range: 50–12.5 ul). Full-length blot is presented in Supplementary M&M. (N = 3; *p < 0.05 vs. sham).

Dot blot analyses performed on culture media collected from NH_4_Cl-treated cells 17 h after DCS (R17) evidenced that NH_4_Cl favors the release of soluble asyn (+ 65%, p < 0.05) and that DCS counteracts this effect bringing back down soluble extracellular asyn levels to control values, thus suggesting that DCS under pathological conditions—here reproduced by the lysosomal inhibition—also affects and modulates the release of soluble asyn (Fig. 8A,B).

## Discussion

Although tDCS has been recognized to improve both motor and non-motor symptoms in PD^2–6,8,10^, the cellular and molecular mechanisms of action still remain elusive. This study was designed to fill this gap using a human neuronal in vitro model with a dopaminergic phenotype to investigate the on-line and off-line effects of DCS at a molecular level on asyn expression, aggregation and degradation.

As a first step, we set up and optimized the experimental protocol to reproduce the current intensity and stimulation time used in the clinical setting, with the limitations that cells can only be subjected to a single stimulation and that only intracellular effects can be studied, without assessing the effects on network modulation and neurotransmitters.

In these conditions, we excluded any cytotoxic effect both during and up to 17 h after stimulation (Supplementary Fig. 1). Importantly, we demonstrated that DCS modifies the protein expression of the different asyn forms, increasing the soluble monomeric asyn and reducing the oligomeric/aggregated forms in a time-dependent manner, displaying the major effects after 17 h from stimulation (off-line effect) (Fig. 1A,B). Accordingly, a recent study performed in the same cell line exposed to an electromagnetic field reported an increase of both monomeric asyn and amyloid precursor protein (APP), paralleled by a reduction of aggregated forms^18^. Furthermore, evidence for a direct effect of the application of a voltage difference on the aggregation status of asyn was already described in an in vitro study^19^.

The quantification of asyn mRNA levels by real-time PCR allowed us to exclude an appreciable effect of DCS on asyn gene transcription (Fig. 1C), thus leading us to investigate if DCS could instead modulate its degradation.

Under standard culture conditions we observed that DCS induces a progressive reduction of both macroautophagy (Fig. 2A,B) and CMA (Fig. 3A,B) effectors (Beclin-1, LC3 and LAMP2A) and an increase of their substrates (p62 and MEF2D), suggesting an overall down-regulation of these catabolic systems. Supporting these results, the assessment of the phosphorylation status –corresponding to activity—of 2 kinasic pathways, Akt and Erk1/2, known to inhibit autophagy via mTOR, confirmed following DCS an overtime increased activation of both pathways, especially Akt (Fig. 2D,E).

Collectively, the results obtained in the first part of this study indicate that, under standard culture conditions, when all cellular homeostatic systems are in a physiological state, the application of DCS increases monomeric and reduces oligomeric asyn forms. This effect is likely ascribable to a mechanic and physical action of DCS that favors the disaggregation of several aggregated proteins including asyn, as already described^19^. The shift of the equilibrium between monomeric and aggregated proteins, including asyn, towards the monomeric/soluble forms is, in our hypothesis, likely to improve the cellular homeostasis, with a feedback effect responsible for a down-regulation of cytoprotective intracellular pathways including autophagy. In fact, macroautophagy and CMA, the two main autophagic pathways involved in asyn catabolism, are known to be specifically involved in the degradation of aggregated or pathologically-modified forms of asyn^20^. Accordingly, gene transcription of the neuroprotective factor BDNF, generally induced under stress conditions, also showed a trend to reduction after DCS (Fig. 1C), and no perturbation of the release of soluble asyn was evidenced.

Due to substantial differences in stimulation protocols and experimental models used, caution should be used when comparing results obtained in this study on the effects of DCS on autophagy with respect to published data. Subtoxic doses of nanosecond pulsed electric fields has been demonstrated in vitro to immediately activate macroautophagy as a possible repair mechanism against stimulation-induced membrane damage, but a prolonged exposure results in a decrease of autophagic markers^21^. It has also been reported that exposure to radiofrequency induces autophagy in pancreatic cancer cells, and that this induction is not detectable in non-tumor cells^22^. Furthermore, in an in vivo study performed in healthy mice, it has been observed that a prolonged exposure to high frequency electromagnetic field leads to an increase of autophagic proteins LC3-II and Beclin-1 and an accumulation of autolysosomes in neuronal cell bodies^23^. An attenuation of macroautophagy activation was also recently described in a rat model of vascular dementia^24^. Notably, all published studies investigated the effect of stimulation on macroautophagy, and no literature data are up to now available on CMA, although CMA dysfunctions has been recognized to play a crucial role in PD pathogenesis^12^.

The analysis of the effects of DCS on the expression of TDP-43, a protein that accumulates in motor neurons of patients with sporadic ALS^25–27^, performed to assess the specificity of the effects observed on asyn, indicated that stimulation has no effect on the protein levels of full-length TDP-43 (Fig. 1A,B). These results are not sufficient to exclude a non-specific effect of DCS possibly favoring the rupture of aggregated protein structures into monomers, and other studies on different aggregate-prone proteins of neuropathological importance, such as beta-amyloid and Tau, are required to solve this issue.

In the second section of our study, the exposure of cells to the mitochondrial inhibitor rotenone allowed us to reproduce a pathological increase of asyn mimicking a synucleinopathy. In this condition, DCS counteracted the marked rotenone-induced increase of monomeric of asyn and also reduced the oligomeric forms (Fig. 5A,B), not acting on asyn gene transcription, but potentiating its degradation via macroautophagy and CMA (increased effectors LC3-II and LAMP2A and reduced substrates p62 and MEF2D) (Fig. 6A–C). Our results fit with recent studies performed in an in vivo mouse model of PD obtained using MPTP in which tDCS has been demonstrated to improve behavioral abilities, reduce MPTP-induced asyn increase by modulating macroautophagy^28^ and increase mitophagy^29^; once again, no data are available on the effect of DCS on CMA pathway.

Furthermore, the use of the lysosomal inhibitor ammonium chloride, that leads to increased asyn protein levels due to a block of lysosomal-dependent degradation, allowed us to conclude that the potentiation of autophagic degradation of asyn is not the only intracellular mechanism induced by DCS. This finding let us to hypothesize that DCS, favoring the solubilization of oligomeric/aggregated asyn, can also stimulate the degradation of asyn via ubiquitin–proteasome system or other non-conventional catabolic pathways mainly involved in the degradation of soluble asyn. According to this hypothesis, we demonstrated that, in presence of a lysosomal inhibition, the release of soluble asyn in the culture medium is increased, and that DCS was able to counteract this abnormal release possibly stimulating different intracellular catabolic pathways (Fig. 8A,B).

Collectively, while under standard culture conditions DCS favors the solubilization of aggregated proteins thus contributing to improve cellular homeostasis, in presence of pathological conditions (here represented by a synucleinopathy) DCS counteracts the accumulation of asyn also potentiating different intracellular mechanisms including the autophagic degradation. Therefore, results obtained in this study support the view that DCS possesses a neuroprotective potential against the toxicity associated to asyn aggregation. Studies are needed to further characterize the intracellular mechanisms elicited by this technique and to verify the specificity of the observed effects for asyn. This knowledge will be useful for ameliorating the identification and selection of patients with PD, other synucleinopathies and proteinopathies that can benefit from this treatment.

Our study has some limitations. First, the direction of the induced electrical field likely differs between in vitro and in vivo conditions; both in animal and human models, the direction strictly depends on the cell type, axon orientation and tDCS montage, and is modified by nearest tissues of different conductivity and resistance^30^. Nonetheless, we are confident that our results are reproducible also in humans basing on similar data recently collected in a model of Alzheimer’s disease (AD), showing that tDCS promotes the degradation and clearance of oligomeric β-amyloid (Aβ_42_), thus improving spatial learning and memory in APP/PS1 transgenic mice^31^. A second potential limitation is about the current density used in our experiments (0.11 mA/cm^2^), higher than the safety values suggested by Bikson and co-workers for human applications^30^. However, the safety range proposed by Bikson and colleagues was mainly based on animal studies; moreover, the brain injury induced by DCS occurs in mice at predicted brain current densities (6.3–13 A/m^2^) that are over an order of magnitude above those produced by conventional tDCS and, consequently, about six-seven times higher than those reported here.

Moreover, although some uncertainties in the translation to human experiments still remain, we are confident that the effects we observed here could be reproduced in vivo by using conventional tDCS at lower current densities (i.e. 0.02 mA/cm^2^). In fact, previous studies describing in vitro after-effects of tDCS had applied higher currents than those used later in humans, confirming most of the mechanisms of action^32^. Finally, given that tDCS effects are both time and intensity-dependent^33,34^, a growing body of literature is moving towards the use of higher current intensities (up to 10.0 mA) than those commonly applied in the clinical practice^35–37^, especially for the prevention and treatment of proteinopathies and neurodegenerative disorders^38^.

Supplementary Fig. 2B and C shows a modeling of the normalized amplitude distribution of the electrical field (E) and the relative current densities (J) for our experiments. Indeed in any conductive medium with uniform conductivity the E and the current density J have a linear correlation and their spatial distribution is therefore identical to less than a scale factor (i.e., the conductivity value).

## Materials and methods

### Cell cultures

Human neuroblastoma SH-SY5Y cells were grown in Dulbecco’s Modified Eagle’s Medium-F12 (EuroClone) supplemented with 10% fetal bovine serum (EuroClone), 100 U/mL penicillin (EuroClone), 100 μg/mL streptomycin (EuroClone) and 2 mM l-glutamine (EuroClone), at 37 °C in an atmosphere of 5% CO_2_ in air.

### Direct current stimulation (DCS)

SH-SY5Y cells were seeded at a density of 80–100,000 cells/mL in Petri dishes (14.5 cm diameter) and, after 24 h, subjected to the stimulation protocol with 1 mA direct current for 20 min, with an initial ramp of current with increasing intensity and a final ramp of 20 s each; stimulation was carried out using 2 synthetic sponges (3 × 3 cm size) soaked in PBS (Phosphate Buffered Saline), immersed in the culture medium in a diametrically opposite position, and connected to a battery-powered stimulator (HDCStim, Newronika) (Supplementary Fig. 2A). Before stimulation, culture medium (50 mL) was added to each dish in order to reach a volume sufficient to ensure the correct passage of current. A modeling of geometry, normalized amplitude distribution of electrical field and relative current densities for our experiments was shown in Supplementary Fig. 2B and C. Constant current flow was measured by an ampere meter, corresponding to a current density of about 0.11 mA/cm^2^ and resulting in a stimulation charge of 1.2 × 10^6^ µA/s (1.2 C), values in line with those commonly used for clinical trials in humans and considered to be safe for translational applications^30,39–42^. During the stimulation, cell dishes were kept without lid at 37 °C in an atmosphere of 5% CO_2_ in air, while the stimulator was kept outside the incubator. For each experiment, sham was obtained using a dedicated dish subjected to the stimulation protocol without current passage.

At the end of each stimulation, cells were observed and imaged under an optical microscope to evaluate a possible effect of the stimulation on cell viability and morphology. Culture medium and cells were collected and stored at − 80 °C for biomolecular analyses.

### Cytotoxicity assay

The effect of DCS on cell viability was assessed by MTT assay at R0, R1 and R17. At each time point, SH-SY5Y cells were incubated with 0.5 mg/ml MTT (Sigma-Aldrich) in standard medium for 45 min at 37 °C in an atmosphere of 5% CO_2_ in air. After cell solubilization with DMSO, absorbance was quantified (wavelength 570 nm) using a multi-mode microplate reader (FLUOstar Omega, BMG LABTECH) and cell viability expressed as % vs. sham group.

### RNA extraction and cDNA synthesis

Total RNA was extracted using the RNeasy Mini kit (Qiagen), according to the manufacturer’s instructions. The RNA concentration was determined spectrophotometrically at 260 nm. RNA (2 µg) was retrotranscribed into cDNA using the SuperScript VILO cDNA Synthesis Kit (Invitrogen) at the following conditions: 10 min at 25 °C and 60 min at 42 °C. The reaction was terminated at 85 °C for 5 min and cDNAs were stored at − 20 °C.

### Real-time quantitative PCR (qPCR)

cDNAs from total RNA (50 ng for beclin-1, LC3, p62, hsc70 and BDNF and 100 ng for asyn, TDP-43, lamp2A, hsp70) were amplified in triplicate in the ABI Prism 7500 HTSequence Detection System (Applied Biosystems). 5 × HOT FIREPol EvaGreen qPCR Mix Plus (ROX) (Solis BioDyne) was used at the following conditions: 95 °C for 15 min, 40 cycles of: 95 °C for 15 s, 62.5 °C for 20 s, 72 °C for20s. The sequences of the primers used (Sigma-Aldrich) are listed in Supplementary Table 1A. To analyze MEF2D mRNA levels, TaqMan Gene Expression Assay (Applied Biosystems, assay ID: Hs00945735_m1; β-actin assay ID: Hs99999903_m1) was used to amplify cDNA (70 ng). For relative quantification of each target vs. β-actin mRNA, the comparative C_T_ method was used.

### Western blotting

Cell pellets were lysed in cell extraction buffer (Invitrogen) supplemented with 1 mM PMSF (Sigma-Aldrich), protease and phosphatase inhibitor cocktail (Sigma-Aldrich) and protein concentration determined by Bradford’s method. Sample lysates were diluted in Laemmli’s loading buffer pH 6.8, denaturated at 95° for 4 min, separated by SDS-PAGE in 4–12% tris glycine gels (Invitrogen) or 15% home-made gels (for LC3I-II) and transferred to nitrocellulose. Blots were blocked for 1 h, incubated overnight at 4 °C with specific primary antibodies (see Supplementary Table 1B) and then with HRP-linked anti-mouse or -rabbit IgG antibody (Sigma-Aldrich) for 1 h. All the details on the methodological approach used to obtain the highest number of targets on the same membrane are specified in Supplementary M&M. Signals were revealed by chemiluminescence, detected using the ImageQuant LAS 4000 (GE Healthcare Life Sciences) imaging system and quantified using the ImageJ software. Protein expression was calculated as ratio between optical densities of the target protein and internal standard (β-actin or GAPDH), and expressed as percentage vs. the mean value of sham group.

### Dot blotting

Dot blot analysis was performed using a Bio-Dot Microfiltration apparatus (Bio-Rad) on culture media. To eliminate insoluble protein fractions and cell debris, culture media were centrifuged at 14,000×*g*, 4 °C for 10 min. 50–12.5 μL of medium were loaded onto a nitrocellulose membrane and deposited by vacuum filtration. Following blocking, the membrane was incubated (overnight at 4 °C) with an anti-α-synuclein primary antibody followed by an HRP-linked secondary antibody (1 h, room temperature). Signals were revealed as above described.

### Statistical analysis

Parametric analyses were used, as all datasets successfully passed the Shapiro–Wilk test for normality (p > 0.05). All data are shown as mean ± standard deviation (SD). Repeated measures ANOVA, followed by Dunnett’s (to compare each group with sham or vehicle group) or Sidak’s (to compare preselected groups) multiple comparison test, was used to assess the significance of differences among groups. Three independent experiments were done for each type of experiment. Statistical analysis was performed using GraphPad Prism 7.04 (GraphPad Software).

## Supplementary Information

Supplementary Information.
