# Author Correction: Direct current stimulation enhances neuronal alpha-synuclein degradation in vitro

**DOI:** 10.1038/s41598-021-88081-2

**Published:** 2021-04-14

**Authors:** Gessica Sala, Tommaso Bocci, Valentina Borzì, Marta Parazzini, Alberto Priori, Carlo Ferrarese

**Affiliations:** 1 Laboratory of Neurobiology, NeuroMI - Milan Center for Neuroscience, School of Medicine and Surgery, University of Milano-Bicocca, via Cadore, 48, 20900 Monza, MB Italy; 2 Centro “Aldo Ravelli” per le Neurotecnologie e le Terapie Neurologiche Sperimentali, Dipartimento di Scienze della Salute, Università degli Studi di Milano and ASST Santi Paolo e Carlo, Milano, Italy; 3 Istituto di Elettronica e di Ingegneria Dell’Informazione e delle Telecomunicazioni (IEIIT), Consiglio Nazionale delle Ricerche (CNR), Milan, Italy; 4 Department of Neurology, ASST-Monza, San Gerardo Hospital, Monza, Italy

Correction to: *Scientific Reports* 10.1038/s41598-021-81693-8, published online 26 January 2021

The original version of this Article contained an error in Affiliation 2, which was incorrectly given as ‘“Aldo Ravelli” Center for Neurotechnology and Experimental Brain Therapeutics, Department of Health Sciences, International Medical School, University of Milan, Milan, Italy’. The correct affiliation is listed below:

Centro “Aldo Ravelli” per le Neurotecnologie e le Terapie Neurologiche Sperimentali, Dipartimento di Scienze della Salute, Università degli Studi di Milano and ASST Santi Paolo e Carlo, Milano, Italy.

The original Article has been corrected.

