# Supplementary material for: Direct current stimulation enhances neuronal alpha-synuclein degradation in vitro
Source: Sci Rep. 2021 Jan 26;11:2197. doi: 10.1038/s41598-021-81693-8 (PMC7838399; doi:10.1038/s41598-021-81693-8)

Title

**Direct current stimulation enhances neuronal alpha-synuclein degradation in vitro.**

Authors

Gessica Sala, Tommaso Bocci, Valentina Borzì, Marta Parazzini, Alberto Priori, Carlo Ferrarese

**Supplementary Table 1**

1. Sequences of primers used (Sigma-Aldrich)

| Target | | Sequence |
| --- | --- | --- |
| α-synuclein | F  R | GCAGCCACTGGCTTTGTCAA |
|  |  | AGGATCCACAGGCATATCTTCCA |
| TDP-43 | F | TGTTTTGCAGCCCTGAATGC |
|  | R | GGAGGACAAAGCCCATTCCA |
| BDNF | F | TGGCTGACACTTTCGAACAC |
|  | R | AGAAGAGGAGGCTCCAAAGG |
| LC3 | F | CAGCATCCAACCAAAATCCC |
|  | R | GTTGACATGGTCAGGTACAAG |
| Beclin-1 | F | ATCTCGAGAAGGTCCAGGCT |
|  | R | CTGTCCACTGTGCCAGATGT |
| p62 | F | CCAGAGAGTTCCAGCACAGA |
|  | R | CCGACTCCATCTGTTCCTCA |
| LAMP2A | F | GCAGTGCAGATGAAGACAAC |
|  | R | AGTATGATGGCGCTTGAGAC |
| HSC70 | F | CAGGTTTATGAAGGCGAGCGTGCC |
|  | R | GGGTGCAGGAGGTATGCCTGTGA |
| β-actin | F | TGTGGCATCCACGAAACTAC |
|  | R | GGAGCAATGATCTTGATCTTCA |

1. List and dilutions of primary antibodies used

| **Target** | **Dilution** |
| --- | --- |
| **α-synuclein** (BD Biosciences) | 1:1000 |
| **TDP-43** (Santa Cruz) | 1:500 |
| **LC3B** (Cell Signaling) | 1:500 |
| **Beclin-1** (Cell Signaling) | 1:1000 |
| **p62** (Cell Signaling) | 1:1000 |
| **Phospho-Akt** (Cell Signaling) | 1:2000 |
| **Phospho-Erk1/2** (Cell Signaling) | 1:2000 |
| **LAMP2A** (Abcam) | 1:900 |
| **HSC70** (Abcam) | 1:3000 |
| **MEF2D** (BD Biosciences) | 1:1000 |
| **GAPDH** (MerckMillipore) | 1:40000 |
| **β-actin** (Sigma-Aldrich) | 1:40000 |

**Supplementary Figure 1**

**Supplementary Figure 2**


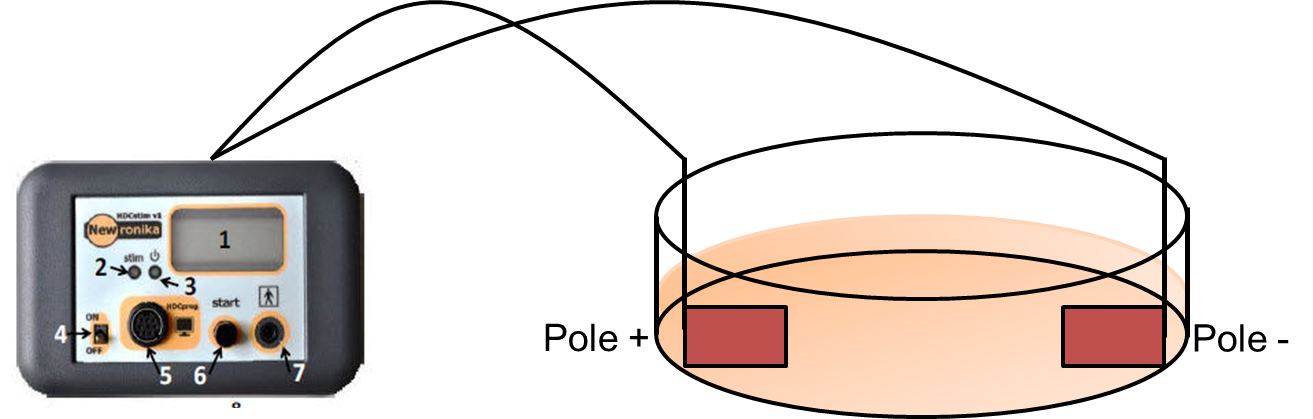


electrical

stimulator


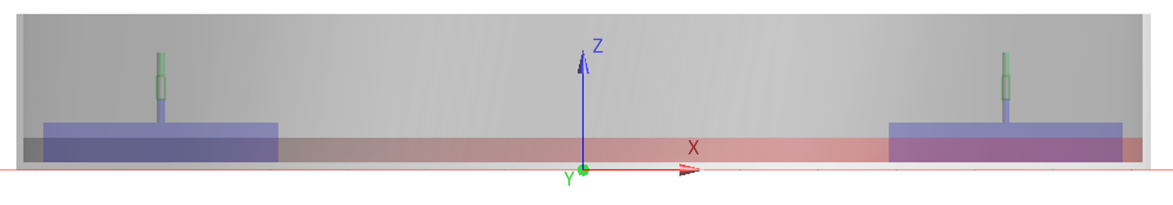


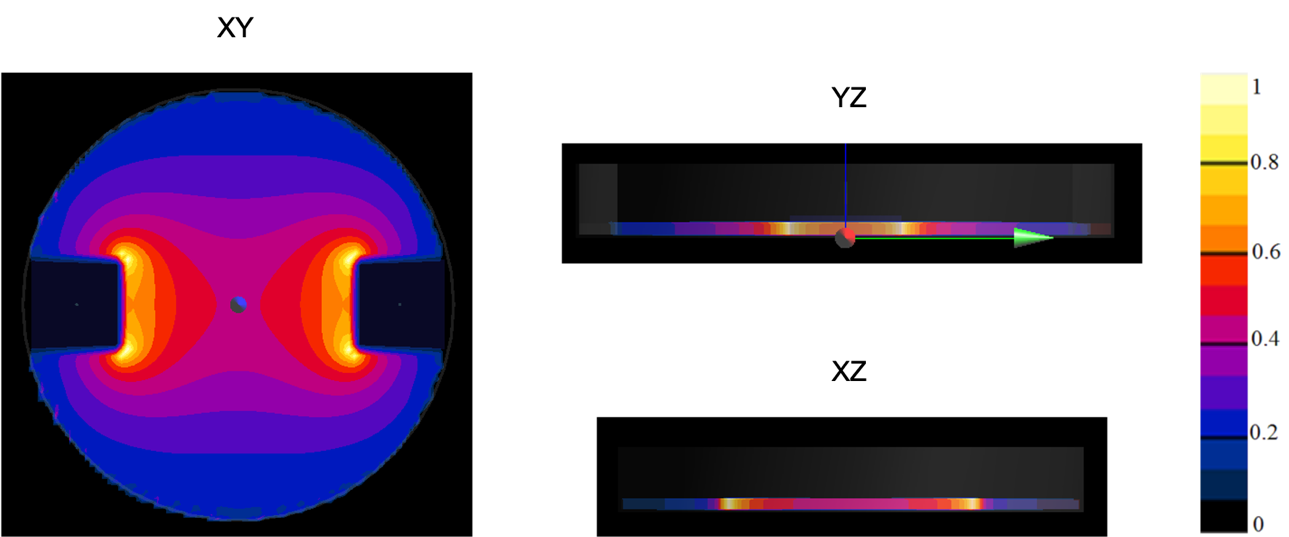


**Supplementary M&M**

All antibodies used in this study was previously used by the corresponding author in Western blot experiments on the same cell line (see published results PMID: 32712350; 27133439; 23984410; 31663379; 29478529).

**Figure 1**

Panel A shows the full-length nitrocellulose membrane with the image of the molecular weight marker and the cut line (dashed line). After immunoblotting, the membrane was horizontally cut above the 24 kDa molecular weight marker band before chemiluminescent detection in order to apply the most suitable chemiluminescent reagent and exposure time optimized based on our experience to better reveal monomeric and oligomeric asyn immunoreactive bands on the same samples. Panel B: merge asyn-molecular weight marker; panel C: asyn immunoreactive bands.


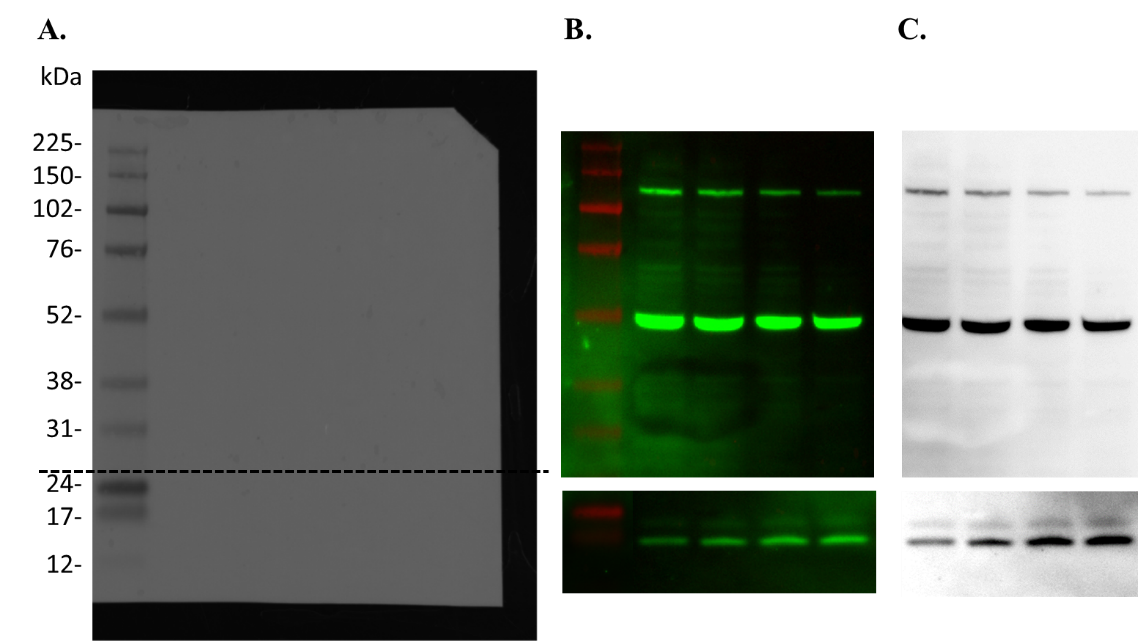


After obtaining asyn signals, the upper part of the membrane was further cut under the 50 kDa asyn band and the obtained part (ranging from >24 to <50 kDa) was hybridized with the anti-TDP-43 antibody and then with the anti-GAPDH antibody, used as internal standard. Below is reported the unprocessed image for TDP-43 and GAPDH immunoreactive bands shown in Figure 1B. Panel A shows the nitrocellulose membrane (cut under 50 kDa and above24 kDa) with the image of the molecular weight marker. Panel B: merge TDP-43-GAPDH-molecular weight marker; panel C: TDP-43 and GAPDH immunoreactive bands.


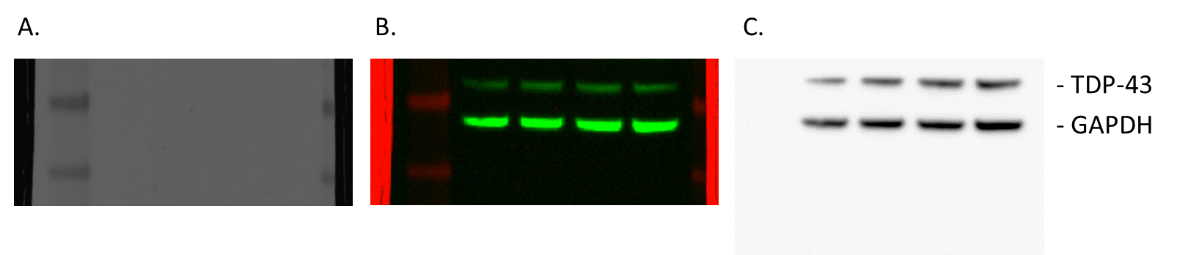


**Figure 2**

Panel A shows the full-length nitrocellulose membrane with the image of the molecular weight marker. The membrane, previously exposed to an anti-asyn antibody followed by a mild stripping, was horizontally cut in correspondence of the 24 kDa molecular weight marker band to eliminate any possible interference of monomeric 19 kDa asyn and then exposed to an anti-p62 antibody. Panel B: merge p62-molecular weight marker; panel C: p62 immunoreactive bands.


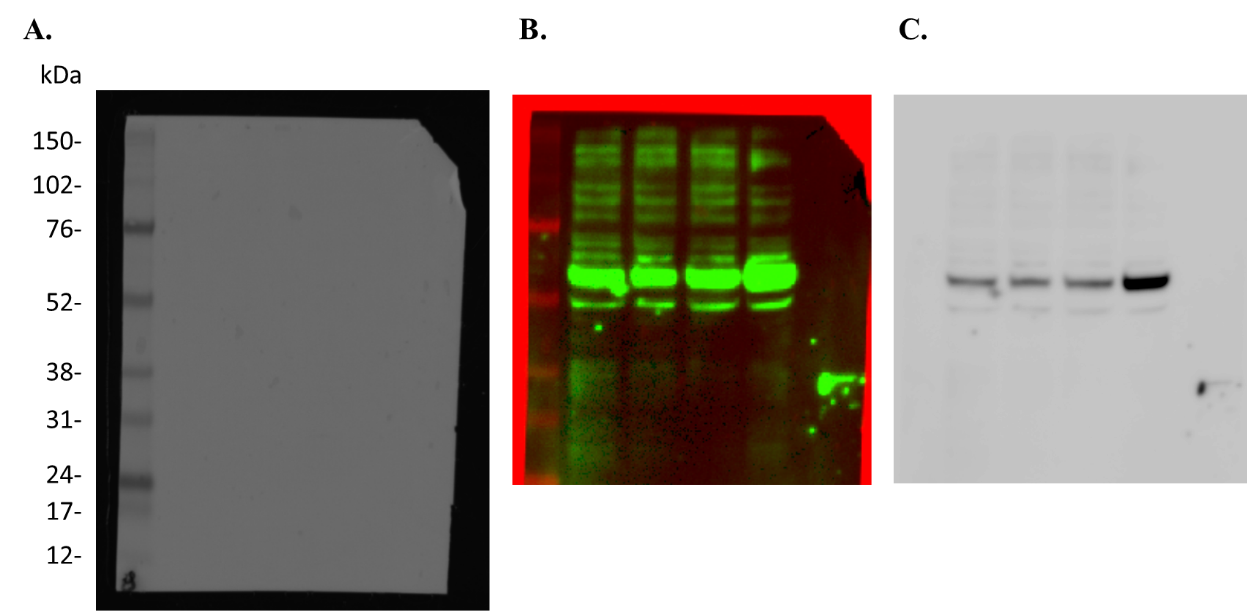


After obtaining p62 signals, the membrane was further cut just under the 50 kDa asyn band and the obtained part (ranging from >24 to <50 kDa) was hybridized with the anti-β-actin antibody, used as internal standard; below is reported the unprocessed image for β-actin immunoreactive bands shown in Figure 2B and used to normalize p62 immunoreactivity (above the β-actin bands are visible faint signals corresponding to the 50 kDa asyn bands not completely eliminated by the cut of the membrane).


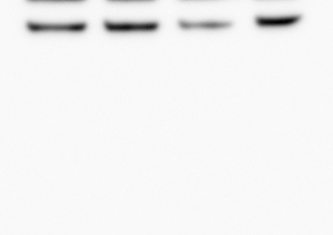


- β-actin

Panel A shows the full-length nitrocellulose membrane with the image of the molecular weight marker. Panel B: merge Beclin-1-molecular weight marker; panel C: Beclin-1 immunoreactive bands.


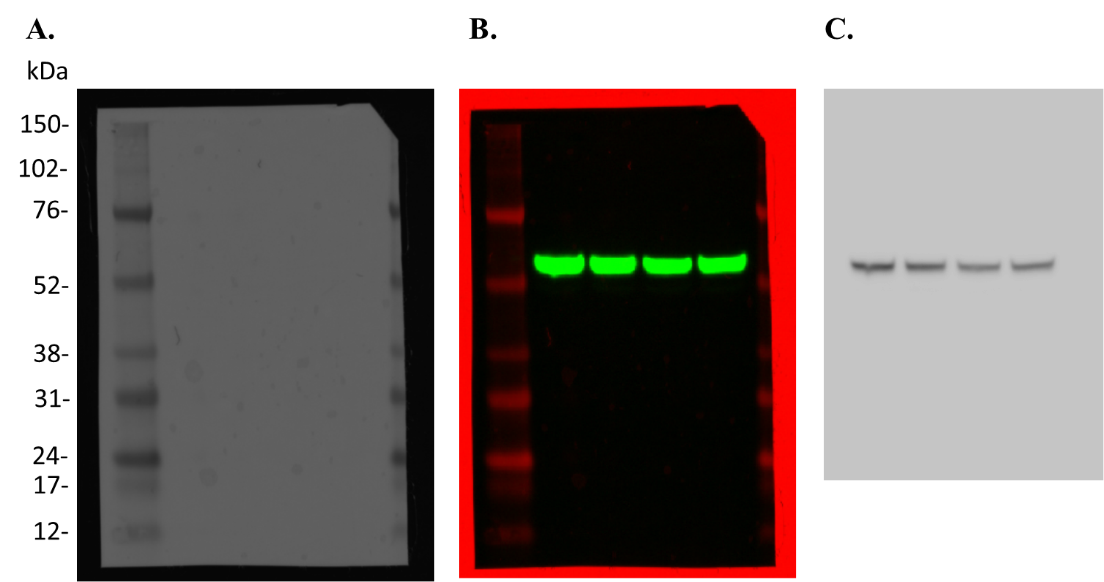


Below is reported the unprocessed image for β-actin immunoreactive bands shown in Figure 2B and used to normalize Beclin-1 immunoreactivity.


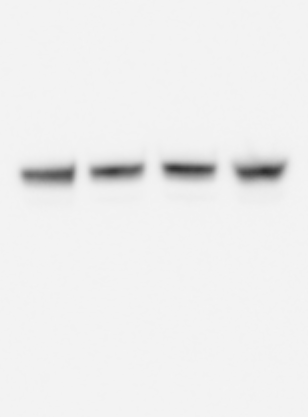


- β-actin

Panel A shows the full-length nitrocellulose membrane with the image of the molecular weight marker. Panel B: merge LC3I/II-β-actin-molecular weight marker; panel C: LC3I/II immunoreactive bands; panel D: β-actin immunoreactive bands (a faint signal corresponding to LC3I/II is also visible).


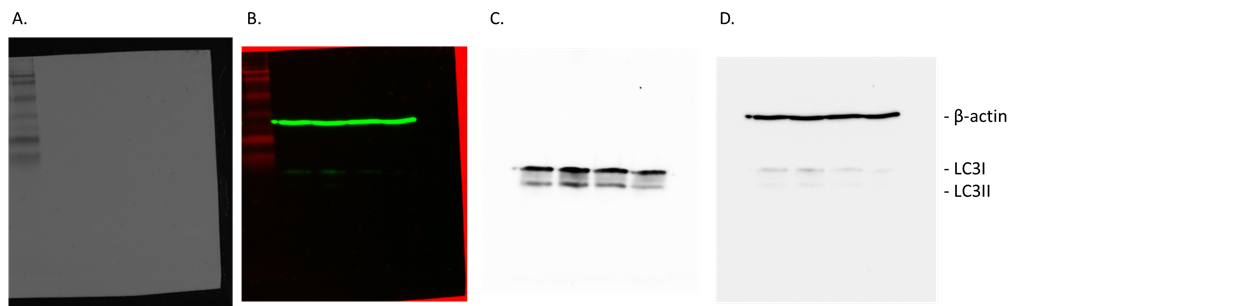


The membrane, previously exposed to an anti-asyn antibody followed by a mild stripping, was horizontally cut under the 50 kDa asyn band and upper the 19 kDa asyn band and then exposed to an anti-phospho- Erk1/2 antibody. Panel A: cropped nitrocellulose membrane with the image of the molecular weight marker; panel B: merge phospho- Erk1/2-molecular weight marker; panel C: phospho- Erk1/2 immunoreactive bands.


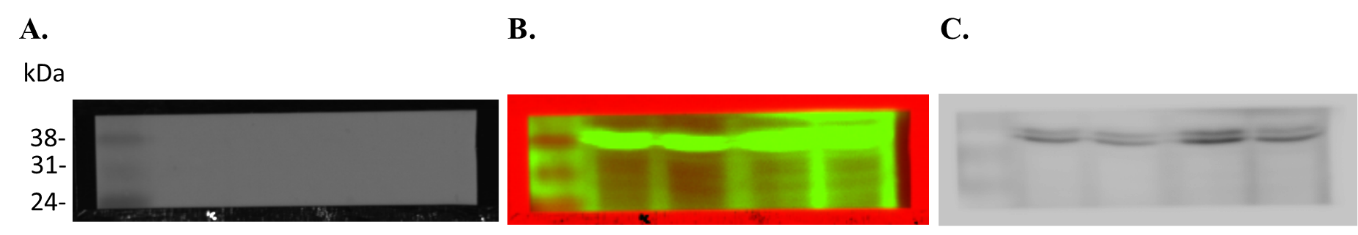


Below is reported the unprocessed image for GAPDH immunoreactive bands shown in Figure 2E and used to normalize p-Erk1/2 immunoreactivity (above the GAPDH bands are visible signals corresponding to p-Erk1/2).


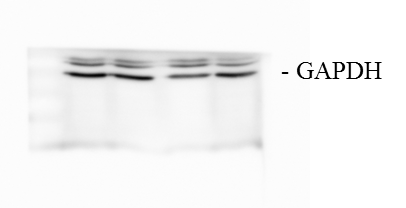


Panel A shows the full-length nitrocellulose membrane with the image of the molecular weight marker. Panel B: merge phosho-Akt-molecular weight marker; panel C: phosho-Akt immunoreactive bands.


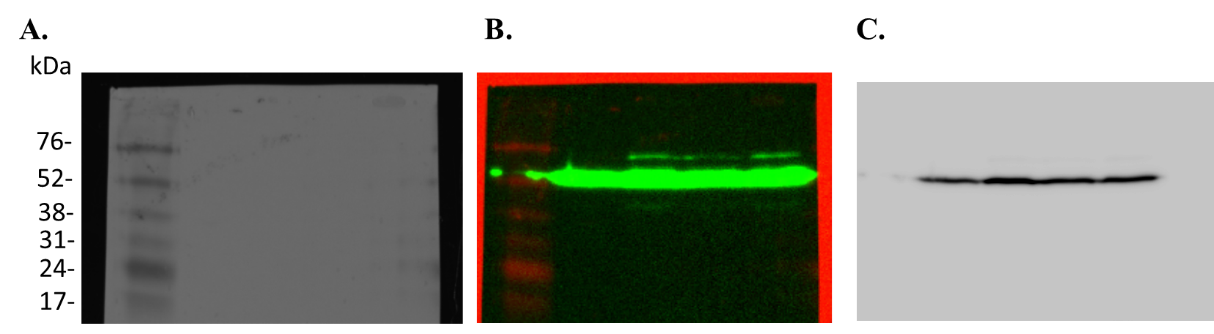


Below is reported the unprocessed image for GAPDH immunoreactive bands shown in Figure 2E and used to normalize p-Akt immunoreactivity (above the GAPDH bands are visible signals corresponding to p-Akt).


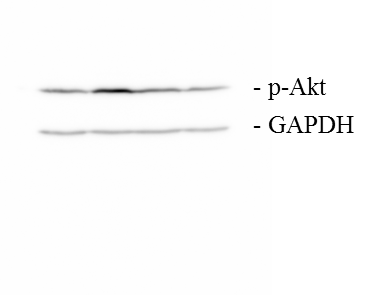


**Figure 3**

The membrane was horizontally cut below the molecular weight marker band corresponding to 102 kDa and then exposed to an anti-LAMP-2A antibody. Panel A: cropped nitrocellulose membrane with the image of the molecular weight marker; panel B: merge LAMP-2A-molecular weight marker; panel C: LAMP-2A immunoreactive bands.


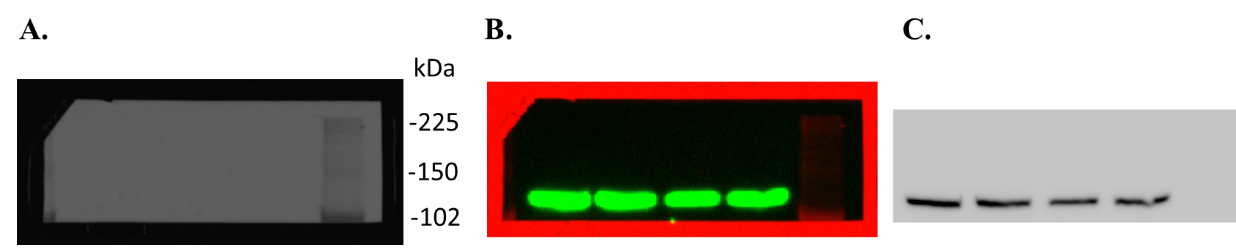


Below is reported the unprocessed image for HSC70 and β-actin immunoreactive bands shown in Figure 3B.


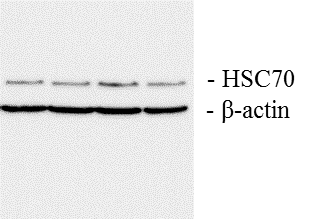


Panel A shows the full-length nitrocellulose membrane with the image of the molecular weight marker. The membrane was then horizontally cut between 24 and 31 kDa based on molecular weight marker bands and then exposed to an anti-MEF2D antibody. Panel B: merge MEF2D-molecular weight marker; panel C: MEF2D immunoreactive bands.


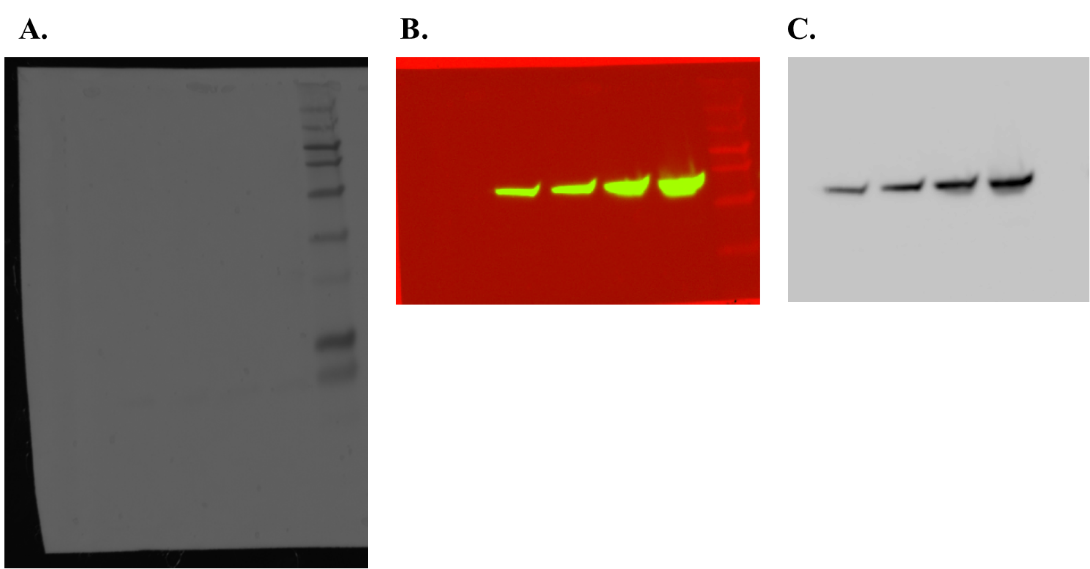


Below is reported the unprocessed image for β-actin immunoreactive bands shown in Figure 3B and used to normalize MEF2D immunoreactivity.


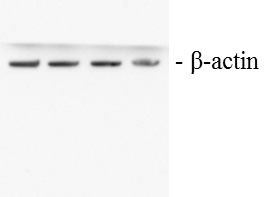


**Figure 4**

Panel A shows the full-length nitrocellulose membrane with the image of the molecular weight marker and the cut line (dashed line). After immunoblotting, the membrane was horizontally cut above the 24 kDa molecular weight marker band before chemiluminescent detection in order to apply the most suitable chemiluminescent reagent and exposure time optimized based on our experience to better reveal monomeric and oligomeric asyn immunoreactive bands on the same samples. Panel B: merge asyn-molecular weight marker; panel C: asyn immunoreactive bands.


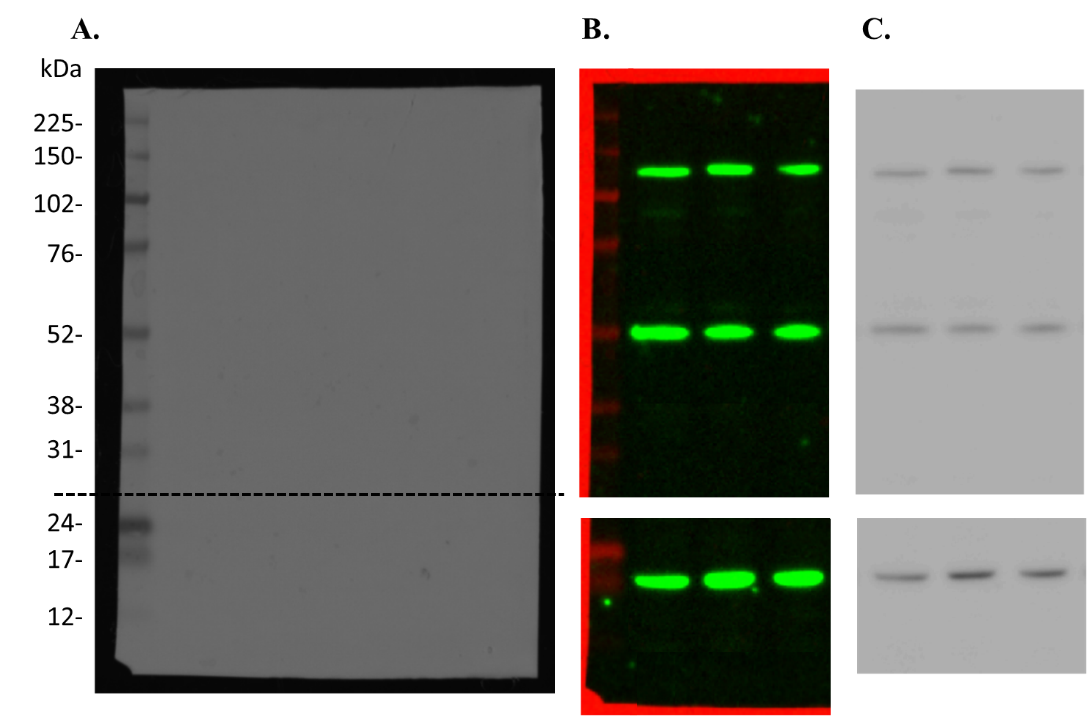


After obtaining asyn signals, the upper part of the membrane was further cut just under the 50 kDa asyn band and the obtained part (ranging from >24 to <50 kDa) was hybridized with the anti-β-actin antibody, used as internal standard; below is reported the unprocessed image for β-actin immunoreactive bands shown in Figure 4B (above the β-actin bands are visible faint signals corresponding to the 50 kDa asyn bands not completely eliminated by the cut of the membrane).


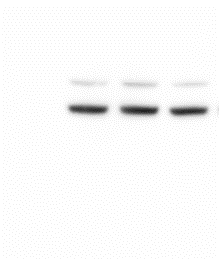


- β-actin

**Figure 5**

Full-length nitrocellulose membrane with the merge of the molecular weight marker and asyn immunoreactive bands (panel A). Panel B: asyn immunoreactive bands; Panel C: improved immunoreactivity for 100 kDa and 19 kDa asyn forms, as displayed in the manuscript in Figure 5B (to obtain these bands, membrane was horizontally cut below the 100 kDa and above the 19 kDa asyn forms in order to apply the most suitable chemiluminescent reagent and exposure time optimized based on our experience to better reveal these bands).


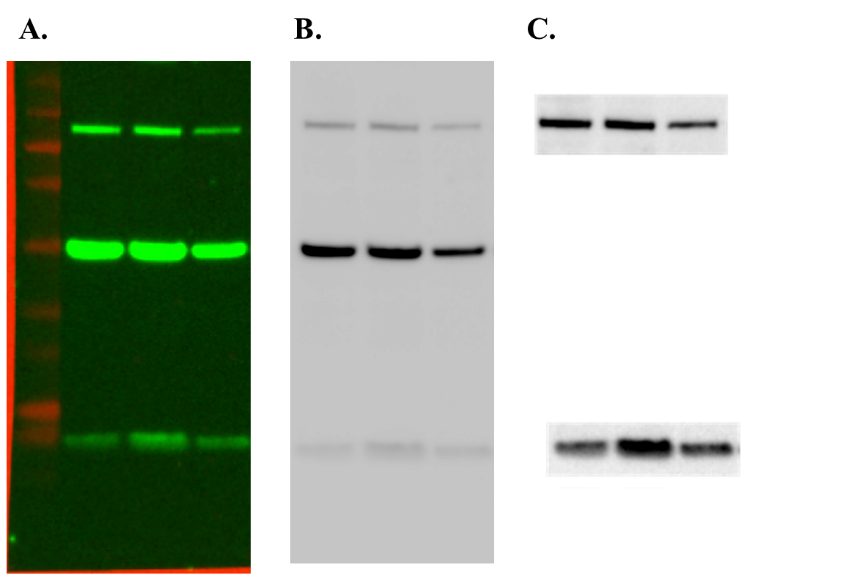


After obtaining asyn signals, the central part of the membrane (ranging from >24 to <100 kDa) was further cut just under the 50 kDa asyn band and the obtained part (ranging from >24 to <50 kDa) was hybridized with the anti-β-actin antibody, used as internal standard; below is reported the unprocessed image for β-actin immunoreactive bands shown in Figure 5B (above the β-actin bands are visible faint signals corresponding to the 50 kDa asyn bands not completely eliminated by the cut of the membrane).


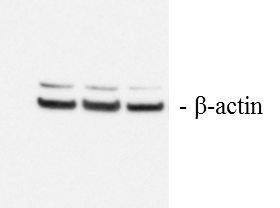


**Figure 6**

Panel A: full-length nitrocellulose membrane of cropped images of p62 and HSC70 shown in Figure 6C. Panel B: the membrane in Panel A was cut above HSC70 bands and below p62 bands and exposed to anti-LAMP-2A (upper part) and MEF2D (lower part) antibodies; cropped images of LAMP2A and MEF2D bands were shown in Figure 6C. Panel C: images of merge LC3-I/II – marker (above) and immunoreactive bands of LC3-I/II (below) in a 15% home-made gel cropped based on the expected molecular weight of target; differently from image shown in Figure 6C further cropped to show 3 lanes in line with other figure panels, 4 lanes are shown in Panel C, with the last one lane on the right representing only a duplicate of the sample loaded in the previous lane..


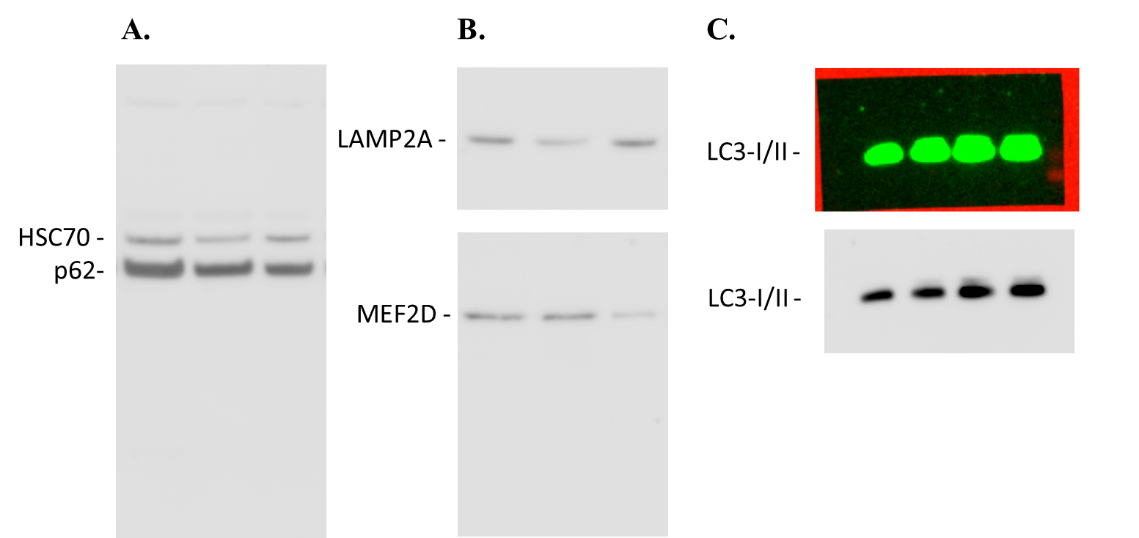


After obtaining signals for p62, HSC70, LAMP2A and MEF2D (shown in panels A and B), the part of the membrane previously hybridized with the anti-MEF2D antibody was hybridized with the anti-β-actin antibody, used as internal standard; below is reported the unprocessed image for β-actin immunoreactive bands shown in Figure 6C (above the β-actin bands are visible faint signals corresponding to MEF2D).


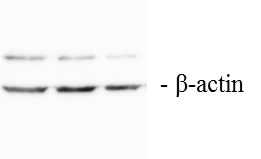


After obtaining signals for LC3-I/II, the upper part of the membrane was hybridized with the anti-Beclin-1 antibody and then with the anti-β-actin antibody, used as internal standard; below are reported the unprocessed image for Beclin-1 and β-actin immunoreactive bands shown in Figure 6C (please note that, differently from image shown in Figure 6C further cropped to show 3 lanes in line with other figure panels, 4 lanes are shown in the image below, with the last one lane on the right representing only a duplicate of the sample loaded in the previous lane).


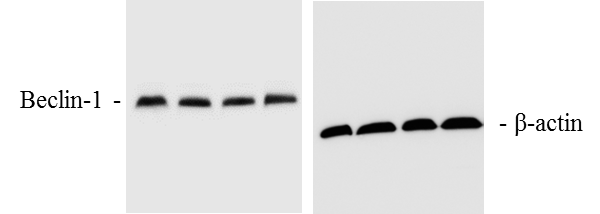


**Figure 7**

The membrane was simultaneously hybridized with an anti-asyn antibody in presence of an anti-β-actin antibody, used as internal standard. Before chemiluminescent detection, the membrane was horizontally cut at 24 kDa (based on the molecular weight marker) in order to apply the most suitable chemiluminescent reagent and exposure time optimized based on our experience to better reveal monomeric and oligomeric asyn immunoreactive bands on the same samples. The immunoreactivity for the internal standard β-actin is shown in the same image.


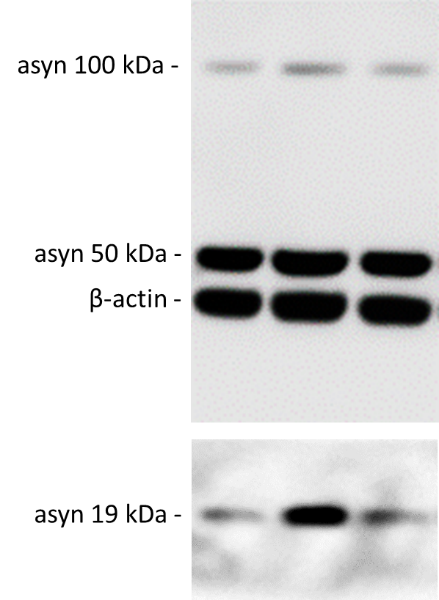


**Figure 8**

Upper panel: original and unprocessed version of the Dot blot image from which Figure 8B was obtained; the edges of the nitrocellulose membrane are visible. Lower panel: improved immunoreactive signal obtained after an adjunctive washing; immunoreactive bands shown in Figure 8B are indicated by the red box.


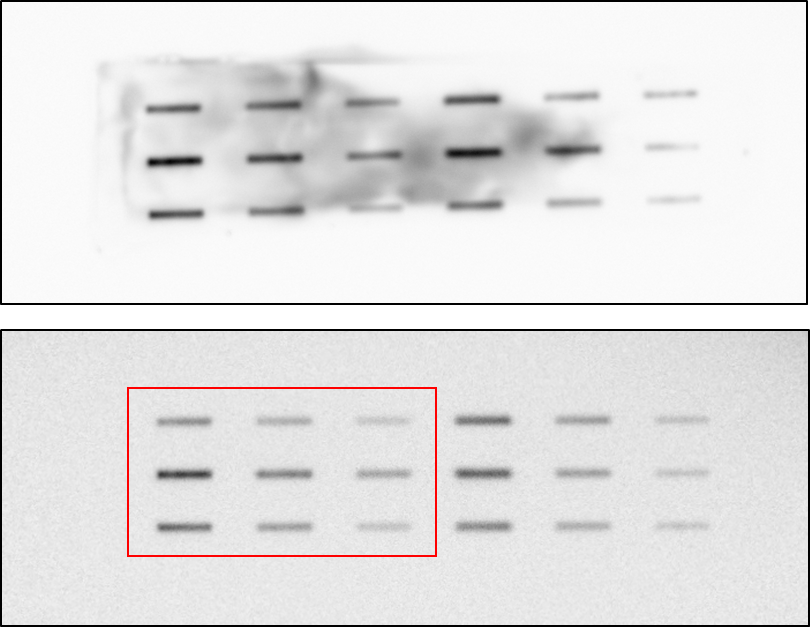

Supplement: Supplementary file 1 — Supplementary Information. [file 41598_2021_81693_MOESM1_ESM.docx]
